# Mechanics of the golf lip out

**DOI:** 10.1098/rsos.250907

**Published:** 2025-11-05

**Authors:** S. John Hogan, Mate Antali

**Affiliations:** ^1^ School of Engineering Mathematics and Technology, University of Bristol, Bristol, UK; ^2^ Department of Applied Mechanics, Szechenyi Istvan Egyetem, Győr, Gyor-Moson-Sopron, Hungary

**Keywords:** golf, putting, lip out

## Abstract

Sometimes, when a golfer attempts to putt a golf ball, it appears to enter the hole, only to re-emerge almost immediately, having undergone an angle of turn around the hole rim that can exceed $180^{\circ }$. We consider the problem from the point of view of mechanics. We show analytically that there are at least two distinct types of *lip out*: the *rim* lip out, where the centre of mass of the golf ball does not fall below the level of the green, and the *hole* lip out where it does. At the heart of both lip outs is a family of degenerate saddle equilibria of the dynamics on the rim (*the golf balls of death*). When perturbed one way, the golf ball executes a rim lip out. When perturbed another way, the golf ball enters the hole, only to re-emerge (provided it does not touch the base of the hole) if it is spinning about an axis perpendicular to the wall of the hole.

## 1. Introduction

Golf has long been the subject of considerable scientific research [1,2]. We are interested in what happens when the ball is on the green, which contains the hole into which the ball must fall. Here, the golfer has to attempt to roll (putt) the ball along the ground and into the hole. Putting, which constitutes 40–45% of all golf shots [3], requires a completely different skill set from other golf strokes. The golf writer Peter Dobereiner (1908−1997) has been quoted as saying, ‘Half of golf is fun, the other half is putting’. The financial importance of putting has been summarized by the South African golfer Bobby Locke (1917−1987) who said: ‘Drive for show, putt for dough’. To get to the green, golfers have to put up with wind, rain, water features, sand traps, rough ground (long grass) and trees while at the same time keeping the ball within bounds. But once on the green, there is another challenge—the lip out. In a lip out, the golfer putts the ball towards the hole. It reaches the rim and appears to fall into the hole, only for it to emerge a moment later at another point on the rim and return onto the green.

The difference between the incoming and outgoing trajectories can be over $180^{\circ }$. The disappointed golfer has not sunk the putt.^1^

We consider this problem from the point of view of mechanics. Our paper is organized as follows. In §2, we discuss the relevant scientific literature. We present our mechanical model of rim and hole motion in §3 with one set of unified variables. In §4, we consider spin-free motion. We show analytically that the separatrices of a family of degenerate saddle equilibria of the rim motion (*the golf balls of death*) divide the phase space dynamics. The golf ball either goes into the hole or experiences a *rim lip out*. In §5, we consider steady-state motion with spin. In §6, we consider the dynamics of rim and hole motion with spin. We show in §7 that, even if the golf ball does enter the hole, in the presence of spin, it can return to the green, in a *hole lip out*, provided it does not touch the bottom of the hole. We discuss our results in §8.

## 2. Previous work

The scientific literature on putting can be roughly divided into two main groups: motion on the rim and motion in the hole.

**Figure 1.**
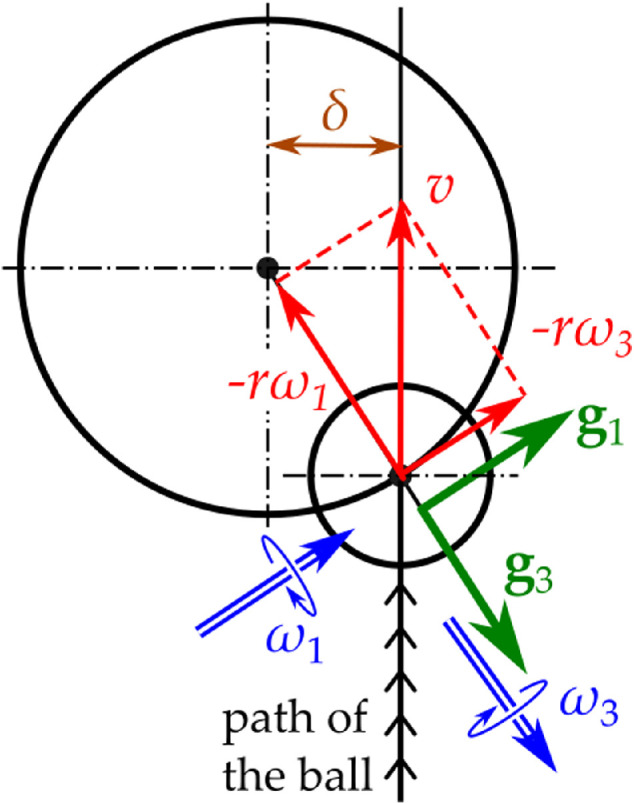
Reparametrization of the rim initial conditions following [8]. By using the ball speed *v* and the impact distance *δ*, the initial conditions of $\omega_{1}(\pi /2)$ and $\omega_{3}(\pi /2)$ at the rim can be found (see equation (3.20)).

Holmes [8] derived equations of motion for a golf ball on the rim of the hole on a green, assuming no axial spin. Outcomes of this study were in terms of the impact distance δ, the lateral displacement of the golf ball trajectory from the centre of the hole and the initial velocity v of the ball at the rim (figure 1). It was shown that a golf ball must travel at 1.626 m s^−1^ or less to be captured in the hole. Theoretical predictions were consistent with experimental results. Subsequently, Hubbard & Smith [4] included axial spin and analysed steady-state rim motions and their stability. They found (numerically) that the boundary in (δ,v) parameter space that separated ‘roll-in’ and ‘roll-out’ trajectories was a barrier that corresponded to initial conditions for a set of near quasi-equilibrium trajectories, with long contact times and large ‘roll-around’ angles. Penner [9] considered the motion of a golf ball on a sloping green and found a modification for the maximum allowed speed for the ball to be captured in the hole. Mahoney & Connaughton [10] reconsidered the case when the golf ball arrives at the hole and is launched towards the opposite rim, improving on the work of Holmes [8]. In 2019, the rules of golf were changed to allow the flagstick to stay in the hole during putting. Subsequently, Kuchnicki [11] included the impact with the flagstick in the Holmes [8] model and concluded that the flagstick is of mixed benefit.

The study of the motion of a rigid sphere on the inside of a cylinder has not always been connected with golf. Routh [12, §225] showed that the horizontal motion of the centre of the sphere is a uniform revolution around the axis of the (perfectly rough) cylinder, and the vertical motion is a harmonic oscillation. Littlewood [13] made the connection with golf, stating [13, p. 46] ‘Golfers are not so unlucky as they think’. Neimark & Fufaev [14, pp. 95−98] showed how to derive the governing equations using a Lagrangian. They showed that the sphere always remained in contact with the cylinder and gave the condition that the sphere rolls without slipping. Gualtieri *et al.* [5] studied the problem both theoretically and experimentally. They demonstrated a (hole) lip out [5, fig. 6] by firing a computer mouse ball at high speed into a transparent vertical cylinder. A simple formulation of the problem was given by Pujol & Pérez [6]. The same authors [7] produced a criterion for the golf ball to emerge from the hole, based on energy conservation, and proved conservation of the vertical component of angular momentum about the point of contact between the golf ball and the hole wall.

There are other papers on different aspects of putting. Bansal & Broadie [15] considered the effect on putting of doubling the hole radius. Drane *et al.* [16] showed both experimentally and numerically that the initial motion of the putt (of any length) is a combination of skidding, sliding and flying. But nearer the hole, the motion becomes pure rolling. They estimated the coefficient of friction μ between the golf ball and the artificial turf to be μ≈0.3. Griffiths *et al.* [17] considered eight different putting surfaces and found that μ∈[0.11,0.40]. In contrast, Hubbard & Smith [4, p. 91] ‘routinely measured ball-green coefficients of friction in the neighbourhood of 0.7’. Daemi *et al.* [18] looked at the effect on putting of a golf ball with an offset centre of mass.

Both authors of the current paper were involved in a study of the related problem of a basketball rolling around the rim of a basketball hoop [19]. There are important similarities and distinct differences between the two problems, which we will highlight below.

## 3. Mechanical model

**Figure 2.**
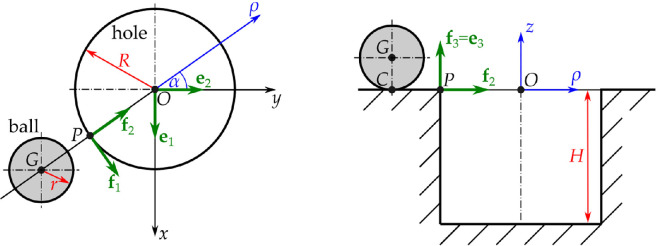
Mechanical model. Left panel: top view of the ball and the hole. Right panel: side view. The Cartesian coordinates x,y,z and the basis vectors $e_{1},e_{2},e_{3}$ are fixed to the hole (ground). The cylindrical coordinates ρ,α,z and basis vectors $f_{1},f_{2},f_{3}$ correspond to the vertical plane containing the centre G of the ball.

Consider the golf ball as a uniform rigid sphere of radius r and dimensionless mass moment of inertia about its centre j (figure 2). On the green, the golf ball moves on a compound rigid surface consisting of three regions. The *ground* is modelled by a horizontal plane, the *hole* is modelled by a vertical cylinder of radius R and depth H and the *rim* is the circle at the intersection of the ground and the hole. In accordance with the relevant rules of golf (see appendix A) and avoiding unrealistic precision, we set r=0.021m, R=0.054m, H=0.102m and m=0.046kg. In addition, we take $j=\frac{2}{5}=0.4$, even though not all golf balls are uniform spheres.^2^ Nevertheless, measurements of j have shown that such an assumption is quite reasonable [7,8]. Our goal is to describe the dynamics in a unified set of variables for the rim and hole motions and to use this formulation to understand lip outs.

### 3.1. Kinematics

We assume that the golf ball rolls without slipping. We ignore air resistance. Initially, we assume that the golf ball is in permanent contact with the surface. As it was shown in a similar problem of the basketball [19], the fundamental structure of the dynamics can be found in the rolling model, and the effects of slip and lift-off are added later as limiting conditions. Similarities between the equations of the golf ball [4] and the basketball [19] are shown in appendix B. In this paper, we will borrow nomenclature from [4] but follow the notation of [19].

#### 3.1.1. Configuration

The calculations are performed in an inertial *reference frame* fixed to the ground, but we can define three *coordinate frames* (sets of orthonormal basis vectors) to simplify some expressions.

**Figure 3.**
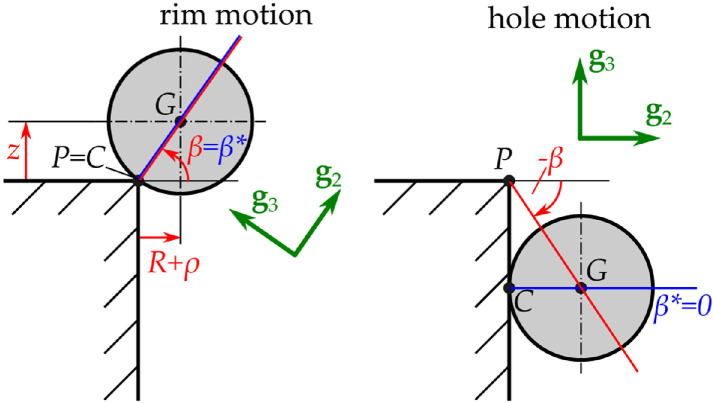
The cases of *rim* and *hole* motion describe the rolling motion of the golf ball in contact with the different regions. The unit vectors $g_{1},g_{2},g_{3}$ are attached to the normal and tangential directions of the contact. The angle of the normal direction is measured by the angle β, while $\beta^{*}$ characterizes the direction of the normal contact direction in the meridian plane.

— Basis vectors $e_{1},e_{2},e_{3}$ are attached to the ground with $e_{3}$ pointing vertically upwards (figure 2).
— Basis vectors $f_{1},f_{2},f_{3}$ are attached to tangential–radial–axial directions of the hole with $f_{2}$ being the radial direction of the centre of the golf ball (the *cylindrical frame*; figure 2).
— Basis vectors $g_{1},g_{2},g_{3}$ are attached to the contact plane of the golf ball with $g_{2}$ being the normal direction (figure 3).

In figures 1 and 2, O is the origin located at the centre of the hole in the plane of the ground; G is the centre of gravity of the golf ball; C is the contact point between the golf ball and the surface; P is the point of the rim in the *meridian plane* containing the golf ball. Using coordinates x,y,z*,* the location of the centre G of the golf ball is given by $r_{OG}=xe_{1}+ye_{2}+ze_{3}$. For the orientation tensor $R\in {SO}^{3}$ of the golf ball, no parametrization (e.g. Euler angles) is needed due to the spherical symmetry of the golf ball. In the cylindrical frame $f_{i}$, we introduce the radial coordinate ρ and the polar angle α (figure 2) where x=ρsin⁡α,y=−ρcos⁡α. Then, the relationship between the bases $e_{i}$ and $f_{i}$ is


(3.1) $$\begin{array}{lcr} & f_{1}=e_{1}\cos\;\alpha +e_{2}\sin\;\alpha ,\;f_{2}=e_{2}\cos\;\alpha -e_{1}\sin\;\alpha ,\;f_{3}=e_{3}. & \end{array}$$

The location of the golf ball centre G in the cylindrical frame becomes


(3.2) $$\begin{array}{lcr} & r_{OG}=\rho f_{2}+zf_{3}, & \end{array}$$

and the point P of the rim in the meridian plane is given by $r_{OP}=-Rf_{2}$.

In the meridian plane spanned by $f_{2}$ and $f_{3}$, for the rim and hole motions, let us express coordinates ρ and z by using a single variable β, the elevation angle of the vector $r_{PG}$, measured from the horizontal plane and defined by tan⁡β=z/(ρ+R) (see figure 3) as follows:

— *rim motion* corresponds to β∈[0,π/2],
— *hole motion* corresponds to β∈(−π/2,0].

The expressions for ρ and z then become


(3.3) $$\begin{array}{r} \rho =\left\{ \begin{array}{ll} -R+r\cos\beta & \text{if}\;\beta \in [0,\pi /2],\\ -R+r & \text{if}\;\beta \in (-\pi /2,0], \end{array}\right. \end{array}$$

(3.4) $$\begin{array}{r} z=\left\{ \begin{array}{ll} r\sin\beta & \text{if}\;\beta \in [0,\pi /2],\\ r\tan\beta & \text{if}\;\beta \in (-\pi /2,0]. \end{array}\right. \end{array}$$

The location of the centre G of the golf ball is now parametrized by the angles α,β. The third spatial coordinate is eliminated by the normal contact constraint between the surface and the golf ball. In the case of the rim motion, this parametrization is equivalent to that of the basketball [19].

We introduce the angle $\beta^{*}$, which is the elevation angle of the vector $r_{CG}$ (see figure 3), and characterizes the direction of the normal contact direction in the meridian plane. It is given by


(3.5) $$\begin{array}{r} \beta^{*}=\left\{ \begin{array}{ll} \beta & \text{if}\;\beta \in [0,\pi /2],\\ 0 & \text{if}\;\beta \in (-\pi /2,0]. \end{array}\right. \end{array}$$

Using $\beta^{*}$, the relationship between the bases $f_{i}$ and $g_{i}$ becomes


(3.6) $$\begin{array}{lrlr} & g_{1} & =f_{1}, & \\ & g_{2} & =f_{2}\cos\;\beta^{*}+f_{3}\sin\;\beta^{*}, & \\ & g_{3} & =-f_{2}\sin\;\beta^{*}+f_{3}\cos\;\beta^{*}. & \end{array}$$

Then, the location of G from the contact point C becomes


(3.7) $$\begin{array}{lcr} & r_{CG}=rg_{2}=r\left(f_{2}\cos\;\beta^{*}+f_{3}\sin\;\beta^{*}\right). & \end{array}$$

#### 3.1.2. Velocity

**Figure 4.**
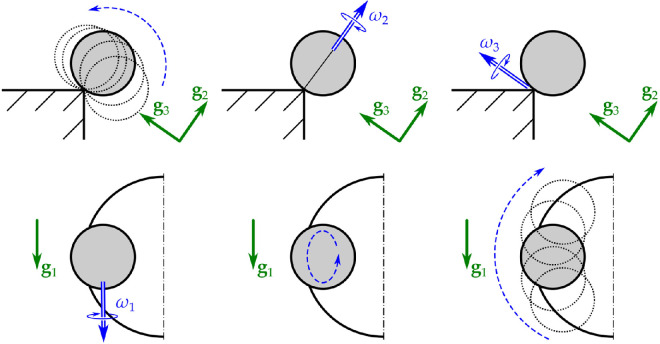
Angular velocity components according to the basis vectors $g_{1},g_{2},g_{3}$, which provide a convenient parametrization of the velocity state of the rolling golf ball. Component $\omega_{1}$ is *pitch*, $\omega_{2}$ is (*axial*) *spin* and $\omega_{3}$ is *roll* [4].


Consider the components of the angular velocity ω in the frame $g_{i}$ (figure 4),

(3.8) $$\begin{array}{lcr} & \omega =\omega_{1}g_{1}+\omega_{2}g_{2}+\omega_{3}g_{3}, & \end{array}$$

which, in the system $f_{i}$, using equation (3.6) becomes


(3.9) $$\begin{array}{lcr} & \omega =\omega_{1}f_{1}+(\omega_{2}\cos\;\beta^{*}-\omega_{3}\sin\;\beta^{*})f_{2}+(\omega_{3}\cos\;\beta^{*}+\omega_{2}\sin\;\beta^{*})f_{3}. & \end{array}$$

Component $\omega_{1}$ is *pitch*, $\omega_{2}$ is (*axial*) *spin* and $\omega_{3}$ is *roll* [4]. The angular velocities of the *rotating coordinate frames* $f_{i}$ and $g_{i}$ are $\omega_{f}=\dot{\alpha }f_{3}$ and $\omega_{g}=\dot{\beta }f_{1}+\dot{\alpha }f_{3}$, respectively, where the dot denotes differentiation with respect to time t. Consequently, the time derivatives of the basis vectors become ${\dot{f}}_{i}=\omega_{f}\times f_{i}$, ${\dot{g}}_{i}=\omega_{g}\times g_{i}$ (i=1,2,3). Then, the velocity of G is given by time derivative of equation (3.2),


(3.10) $$\begin{array}{lcr} & v_{G}={\dot{r}}_{OG}=-\dot{\alpha }\rho f_{1}+\dot{\rho }f_{2}+\dot{z}f_{3}. & \end{array}$$

Alternatively, from the rolling condition $v_{C}=0$ at the contact point, the velocity of G can be expressed as


(3.11) $$\begin{array}{lcr} & v_{G}=\omega \times r_{CG}=-\omega_{3}rg_{1}+\omega_{1}rg_{3}. & \end{array}$$

Then, by comparing equations (3.10) and (3.11) and using equation (3.6), we find


(3.12) $$\dot{\alpha }\rho =\omega_{3}r,\;\;\dot{\rho }=-\omega_{1}r\sin\beta^{*},\;\;\dot{z}=\omega_{1}r\cos\beta^{*}.$$

By using equations (3.3)–(3.5) with equation (3.12), the time derivatives of α and β are given by

— rim motion:

(3.13) $$\dot{\alpha }=\frac{-\omega_{3}r}{R-r\cos\beta },\;\;\dot{\beta }=\omega_{1},$$

— hole motion:

(3.14) $$\dot{\alpha }=\frac{-\omega_{3}r}{R-r},\;\;\dot{\beta }=\omega_{1}\cos^{2}\beta .$$

#### 3.1.3. Acceleration

From the time derivative of equation (3.10), we get the acceleration of G,


(3.15) $$\begin{array}{lcr} & a_{G}=(-\overset{"}{\alpha }\rho -2\dot{\alpha }\dot{\rho })f_{1}+(\overset{"}{\rho }-{\dot{\alpha }}^{2}\rho )f_{2}+\overset{"}{z}f_{3}, & \end{array}$$

and from the time derivative of equation (3.12), we get the equations


(3.16) $$\begin{array}{rl} -\ddot{\alpha }\rho -\dot{\alpha }\dot{\rho } & =-{\dot{\omega }}_{3}r,\\ \ddot{\rho } & =-{\dot{\omega }}_{1}r\sin\beta^{*}-\omega_{1}{\dot{\beta }}^{*}r\cos\beta^{*},\\ \ddot{z} & ={\dot{\omega }}_{1}r\cos\beta^{*}-\omega_{1}{\dot{\beta }}^{*}r\sin\beta^{*}. \end{array}$$

Substitution of equation (3.16) into equation (3.15) leads to


(3.17) $$\begin{array}{lcr} & a_{G}=(-{\dot{\omega }}_{3}r-\dot{\alpha }\dot{\rho })g_{1}+(-\omega_{1}{\dot{\beta }}^{*}r-{\dot{\alpha }}^{2}\rho \;\cos\;\beta^{*})g_{2}+({\dot{\omega }}_{1}r+{\dot{\alpha }}^{2}\rho \;\sin\;\beta^{*})g_{3}. & \end{array}$$

Finally, from equation (3.8), the angular acceleration is given by


(3.18) $$\begin{array}{lcr} & \varepsilon =\dot{\omega }=({\dot{\omega }}_{1}+\omega_{3}\dot{\alpha }\sin\;\beta^{*}-\omega_{2}\dot{\alpha }\cos\;\beta^{*})g_{1}+({\dot{\omega }}_{2}-\omega_{3}{\dot{\beta }}^{*}+\omega_{1}\dot{\alpha }\cos\;\beta^{*})g_{2} & \\ & +({\dot{\omega }}_{3}+\omega_{2}{\dot{\beta }}^{*}-\omega_{1}\dot{\alpha }\sin\;\beta^{*})g_{3}. & \end{array}$$

#### 3.1.4. Ground motion

On the green, when the golfer strikes the golf ball, it must initially slide, possibly bouncing as it goes [16]. But as it approaches the hole, the golf ball invariably begins to roll without slipping, possibly with some spin. Our choice of basis vectors means that^3^ $v_{G}=-vg_{3}$.

The golf ball arrives at the rim with non-zero speed v, but it is seldom travelling directly towards the centre of the hole. Instead, its velocity vector is offset by an amount δ∈[0,R], called the *impact distance* [4,8] (figure 1). We refer to the non-dimensional quantity δ/R as the *impact parameter*.

The speed v and the *impact distance* δ are defined by


(3.19) $$v=r\sqrt{\omega_{1}^{2}(\pi /2)+\omega_{3}^{2}(\pi /2)},\;\;\delta =R\frac{\omega_{3}(\pi /2)}{\sqrt{\omega_{1}^{2}(\pi /2)+\omega_{3}^{2}(\pi /2)}}.$$

The inverse transformation gives us initial conditions^4^ for $\omega_{1,3}$ at the rim [4, eq. (11)]


(3.20) $$\begin{array}{lrr} & \omega_{1}(\pi /2)=-\frac{v\sqrt{R^{2}-\delta^{2}}}{rR},\;\omega_{3}(\pi /2)=-\frac{v\delta }{rR}. & \end{array}$$

### 3.2. Newton–Euler equations of motion

Due to the symmetries of the problem, it is convenient to derive the equations of motion of the golf ball by using the Newton–Euler equations of rigid body dynamics. Any frictional torque and other dissipation effects are neglected for the moment. The golf ball of mass m is subjected to the gravitational force


(3.21) $$\begin{array}{lcr} & F_{G}=-mg\;\sin\;\beta^{*}g_{2}-mg\;\cos\;\beta^{*}g_{3}. & \end{array}$$

where $g=9.81\;m{\;s}^{-2}$ is the acceleration due to gravity. We consider a force acting at the contact point in the form


(3.22) $$\begin{array}{lcr} & F_{C}=F_{1}g_{1}+F_{2}g_{2}+F_{3}g_{3}. & \end{array}$$

Then, the Newton–Euler equations of the golf ball are given by


(3.23)maG=FC+FG,(3.24)jmr2ε=rGC×FC.

By substituting equations (3.17), (3.18) and equations (3.21), (3.22) into equations (3.23), (equation 3.24) and using equation (3.12), we find (see appendix D)


(3.25) $$\begin{array}{lcr} & \begin{array}{rl} (1+j)r^{2}{\dot{\omega }}_{1} & =-(1+j){\dot{\alpha }}^{2}\rho r\;\sin\;\beta^{*}+jr^{2}\omega_{2}\dot{\alpha }\cos\;\beta^{*}-rg\;\cos\;\beta^{*},\\ jr^{2}{\dot{\omega }}_{2} & =jr^{2}\omega_{3}{\dot{\beta }}^{*}-jr^{2}\omega_{1}\dot{\alpha }\cos\;\beta^{*},\\ (1+j)r^{2}{\dot{\omega }}_{3} & =(1+j)r^{2}\dot{\alpha }\omega_{1}\sin\;\beta^{*}-jr^{2}\omega_{2}{\dot{\beta }}^{*}, \end{array} & \end{array}$$

where the normal contact forces $F_{1},F_{2},F_{3}$ are given by


(3.26) $$\begin{array}{lcr} & \begin{array}{rl} F_{1} & =\frac{mjr\omega_{2}{\dot{\beta }}^{*}}{1+j},\\ F_{2} & =mg\;\sin\;\beta^{*}-m\omega_{1}{\dot{\beta }}^{*}r-m{\dot{\alpha }}^{2}\rho \;\cos\;\beta^{*},\\ F_{3} & =\frac{jmg\;\cos\;\beta^{*}}{1+j}+\frac{jmr^{2}\omega_{2}\omega_{3}\cos\;\beta^{*}}{(1+j)\rho }. \end{array} & \end{array}$$

We assume rolling without slipping and so components $F_{i}$ in equation (3.26) are computed as *constraint forces*. Then the dynamic condition for rolling is


(3.27) $$\begin{array}{lcr} & \sqrt{F_{1}^{2}+F_{3}^{2}}\leq \mu F_{2}. & \end{array}$$

Otherwise, the golf ball starts slipping. To ensure that the golf ball does not separate from the surface, we require^5^ $F_{2}\geq 0$, already included in the stricter condition (equation (3.27)).

The equations of rim and hole motion can be obtained by substituting equations (3.5) and (3.12) into equation (3.25). The corresponding normal forces can be obtained similarly from equation (3.26).

#### 3.2.1. Equations of rim motion

The rim motion occurs when β∈[0,π/2]. From equations (3.3) and (3.5), we have $\beta^{*}=\beta$ and ρ=−R+rcos⁡β. Then, the equations of the rim motion become


(3.28)ω˙1=(1+j)rω32sin⁡β−jrω2ω3cos⁡β(1+j)(R−rcos⁡β)−gcos⁡βr(1+j),(3.29)ω˙2=Rω1ω3R−rcos⁡β,(3.30)ω˙3=−rω1ω3sin⁡βR−rcos⁡β−jω1ω21+j,(3.31)α˙=−rω3R−rcos⁡β,(3.32)β˙=ω1.

The contact forces of the rim motion are


(3.33) $$\begin{array}{lcr} & \begin{array}{rl} F_{1} & =\frac{jmr\omega_{1}\omega_{2}}{1+j},\\ F_{2} & =mg\;\sin\;\beta -m\omega_{1}^{2}r+\frac{mr^{2}\omega_{3}^{2}\cos\;\beta }{R-r\;\cos\;\beta },\\ F_{3} & =\frac{jmg\;\cos\;\beta }{1+j}-\frac{jmr^{2}\omega_{2}\omega_{3}\cos\;\beta }{\left(1+j)(R-r\;\cos\;\beta \right)}. \end{array} & \end{array}$$

The equations of the rim motion are equivalent to those of the basketball [19, eqs. (30)–(35)], in the case of pure rolling on a hoop of zero radius.

#### 3.2.2. Equations of hole motion

The hole motion occurs when β∈(−π/2,0]. From equations (3.3) and (3.5), we have $\beta^{*}=0$ and ρ=−R+r. Then, the equations of hole motion [5–7,12] become


(3.34)ω˙1=−jrω2ω3(1+j)(R−r)−gr(1+j),(3.35)ω˙2=rω1ω3R−r,(3.36)ω˙3=0,(3.37)α˙=−rω3R−r,(3.38)β˙=ω1cos2⁡β.

The contact forces of the hole motion are


(3.39) $$\begin{array}{lcr} & F_{1}=0,\;F_{2}=\frac{m\omega_{3}^{2}r^{2}}{R-r},\;F_{3}=\frac{jmg}{1+j}-\frac{jmr^{2}\omega_{2}\omega_{3}}{\left(1+j)(R-r\right)}. & \end{array}$$

### 3.3. Equations of spin-free motion

Previous authors [4,8] have considered motion (on the rim) in the absence of rotation in the $g_{2}$ direction, that is, $\omega_{2}(t)\equiv 0$, the so-called *spin-free* (or *no-spin*) motion. To obtain the equations of spin-free motion, it is not enough just to set $\omega_{2}(t)=0$. For the rim motion, that would mean ${\dot{\omega }}_{2}=0$ in equation (3.29), and so we must take either $\omega_{1}(t)=0$ or $\omega_{3}(t)=0$, clearly a trivial result. Similarly, for the hole motion setting, ${\dot{\omega }}_{2}=0$ in equation (3.35) implies that $\omega_{3}(t)=0$ (taking $\omega_{1}(t)=0$ gives a contradiction). Instead, we must rederive the equations of spin-free motion for both the rim and the hole. We show that these equations can only be correctly derived assuming a specific form^6^ of *dry friction (pivoting) torque* $M_{C}$ given by


(3.40) $$M_{C}=M_{2}g_{2}.$$

We will assume that the friction forces $F_{1},F_{3}$ and the friction torque $M_{2}$ are not coupled but act independently.^7^ Then, the rolling condition equation (3.27) consists of two separate dynamic conditions: no-slip and no-spin in the form


(3.41) $$\sqrt{F_{1}^{2}+F_{3}^{2}}\leq \mu F_{2},\;\;|M_{2}|\leq \eta (F_{2})\mu F_{2}.$$

In equation (3.41), $\eta (F_{2})$ can be estimated, e.g. from the parabolic Hertz contact pressure distribution.^8^

Consider the Newton–Euler equations (3.23) and (3.24). By adding the friction torque equation (3.40), equation (3.23) remains unchanged, while equation (3.24) becomes


(3.42) $$\begin{array}{lcr} & jmr^{2}\varepsilon =r_{GC}\times F_{C}+M_{C}, & \end{array}$$

Compared with equation (3.25), only the second component changes, and we get (since $\omega_{2}=0$)


(3.43) $$\begin{array}{lcr} & 0=jr^{2}\omega_{3}{\dot{\beta }}^{*}-jr^{2}\omega_{1}\dot{\alpha }\cos\;\beta^{*}+M_{2}/m. & \end{array}$$

#### 3.3.1. Equations of spin-free rim motion

Let us solve equations (3.23) and (3.42) by considering the rim motion from equations (3.12) and (3.5). Then, the equations of spin-free rim motion are


(3.44)ω˙1=rω32sin⁡β(R−rcos⁡β)−gcos⁡βr(1+j),(3.45)ω˙3=−rω1ω3sin⁡βR−rcos⁡β,(3.46)α˙=−rω3R−rcos⁡β,(3.47)β˙=ω1,

and


(3.48) $$\begin{array}{lcr} & \begin{array}{rl} F_{1} & =0,\;F_{2}=mg\;\sin\;\beta -m\omega_{1}^{2}r+\frac{mr^{2}\omega_{3}^{2}\cos\;\beta }{R-r\;\cos\;\beta },\\ F_{3} & =\frac{jmg\;\cos\;\beta }{1+j},\;M_{2}^{\text{rim}}=-\frac{mjr^{2}R\omega_{1}\omega_{3}}{R-r\;\cos\;\beta }. \end{array} & \end{array}$$

#### 3.3.2. Equations of spin-free hole motion

Similarly, the equations of spin-free hole motion are


(3.49)ω˙1=−gr(1+j),(3.50)ω˙3=0,(3.51)α˙=−rω3R−r,(3.52)β˙=ω1cos2⁡β.

The contact forces of the hole motion are


(3.53) $$\begin{array}{lcr} & F_{1}=0,\;F_{2}=\frac{m\omega_{3}^{2}r^{2}}{R-r},\;F_{3}=\frac{jmg}{1+j}-\frac{jmr^{2}\omega_{2}\omega_{3}}{\left(1+j)(R-r\right)},\;M_{2}^{\text{hole}}=-\frac{mjr^{3}\omega_{1}\omega_{3}}{R-r}. & \end{array}$$

Note that neither $M_{2}^{\text{rim,hole}}$ is proportional to $F_{2}$ [4].

### 3.4. Conservation of energy

The total energy E of the motion is conserved, both on the rim and in the hole. We have


(3.54) $$\begin{array}{lcr} & E=\frac{1}{2}mjr^{2}\omega_{2}^{2}+\frac{1}{2}m(1+j)r^{2}(\omega_{1}^{2}+\omega_{3}^{2})+U, & \end{array}$$

where the potential energy U=mgrsin⁡β on the rim [19, eq. (43)], and U=mgz in the hole [7, eq. (17)]. It will prove useful to consider the relative contributions to the total energy. Let $E_{1,2,3,\text{pot}}$ be defined as


(3.55) $$\begin{array}{lcr} & E_{n}=\frac{\frac{1}{2}m(1+j)r^{2}\omega_{n}^{2}}{E},\;(n=1,3),\;E_{2}=\frac{\frac{1}{2}mjr^{2}\omega_{2}^{2}}{E},\;E_{\text{pot}}=\frac{U}{E}. & \end{array}$$

Then, $E_{1,2,3}$ are the relative energies due to pitch, spin and roll, respectively, and $E_{\text{pot}}$ is the relative potential energy. The phase space has some interesting symmetries^9^ (see also [19]).

## 4. Spin-free motion

In this section, we assume that the golf ball has zero spin ($\omega_{2}\equiv 0$) throughout its motion. We will uncover the mechanism for the *rim lip out*, using analytic expressions, and so pave the way for our subsequent study of motion with spin.

For spin-free rim motion, we shall consider steady states and use energy conservation to understand the dynamics. We will derive exact expressions for the boundaries in $\left(\frac{\delta }{R},v\right)$-space, which separate different behaviours, verifying earlier numerical work [8, fig. 12]. For spin-free hole motion, we solve the equations explicitly.

### 4.1. Steady-state spin-free rim motion

We consider steady-state spin-free rim motions. Since the α motion is cyclic, we consider only the three-dimensional dynamics of $x(t)\equiv (\beta ,\omega_{1},\omega_{3}{)}^{T}$.

#### 4.1.1. Steady-state solution

The steady-state solutions of equations (3.44), (3.45) and (3.47) are


(4.1) $$\begin{array}{lcr} & x_{ern}=(\beta_{e},0,\omega_{3e}(\beta_{e}){)}^{T} & \end{array}$$

where^10^


(4.2) $$\begin{array}{lcr} & \omega_{3e}(\beta_{e})=\pm \sqrt{\frac{g\;\cot\;\beta_{e}(R-r\;\cos\;\beta_{e})}{r^{2}(1+j)}}. & \end{array}$$

The steady solutions form a one-parameter family in $\left(\beta ,\omega_{1},\omega_{3}\right)$ phase space. For any $\beta_{e}\in \left(0,\pi /2\right]$*,* there exists an angular velocity component $\omega_{3e}(\beta_{e})$ given by equation (4.2) that maintains a steady-state spin-free motion, rolling around the rim with zero pitch, which we call the *golf balls of death.*^11^ Substitution of the steady solution equation (4.1) into equation (3.48) gives $M_{2}^{\text{rim}}=0$. Hence, the steady-state spin-free rim motion can be maintained without the need for a frictional torque.

The practical occurrence of the steady-state spin-free rim solution (equations (4.1) and (4.2)) is limited by several factors: *stability loss*, the effects of *slipping and separation* from the rim and the maximum available *kinetic energy*.

#### 4.1.2. Limiting conditions

*Stability.* After linearization of equations (3.44), (3.45) and (3.47) around the steady solutions equation (4.2), the resulting characteristic polynomial for the motion eigenvalue λ becomes


(4.3) $$\lambda (\lambda^{2}-h(\beta_{e}))=0,$$

where


(4.4) $$\begin{array}{lcr} & h(\beta_{e})=g\frac{(R-r\;\cos\;\beta_{e})-3r\;\cos\;\beta_{e}\sin^{2}\;\beta_{e}}{(1+j)r(R-r\;\cos\;\beta_{e})\;\sin\;\beta_{e}}. & \end{array}$$

It is straightforward to show that $h(\beta_{e})>0,\;\forall \;\beta_{e}\in [0,\frac{\pi }{2}]$. Hence, we have a single zero eigenvalue and two real eigenvalues of opposite sign, and $\beta_{e}$ is a (degenerate) saddle.

*Slipping and separation.* For no-slip and no-spin, the condition equation (3.41) becomes


(4.5) $$\begin{array}{lcr} & \frac{j\;\cos\;\beta_{e}\sin\;\beta_{e}}{\left(1+j\;\sin^{2}\;\beta_{e}\right)}\leq \mu . & \end{array}$$

The minimum value of the left-hand side of equation (4.5) occurs when $\cos\;\beta_{e}=\sqrt{\frac{1+j}{2+j}}=\sqrt{\frac{7}{12}}$, given by $\mu_{\text{min}}=\frac{j}{2\sqrt{1+j}}=\frac{1}{\sqrt{35}}\approx 0.17$ when j=2/5. If $\mu >\mu_{\text{min}}$, then no-slip can occur for the steady-state spin-free motions. In practice, it appears from the literature (see §2) that $\mu >\mu_{\text{min}}$ in general.^12^

*Kinetic energy.* The condition of maximal available kinetic energy for the steady-state spin-free motion can be written from equation (3.54) in the form


(4.6) $$\begin{array}{lcr} & (1+j)r^{2}\omega_{3e}^{2}\leq v^{2}. & \end{array}$$

Using equation (4.2), it can be shown that equation (4.6) provides a lower limit for $\beta_{e}$. For example, when $v=2\;ms^{-1}$, the steady solutions exist for $\beta_{e}⪆0.03\approx 2^{\circ }$.

### 4.2. Phase-plane dynamics of spin-free rim motion

We show that the equation for conservation of total energy of the spin-free rim motion $E_{\text{rim}}$ gives the dynamics of spin-free rim motion explicitly. From equation (3.54) with $\omega_{2}\equiv 0$ we have


(4.7) $$\begin{array}{lrlr} & E=E_{\text{rim}} & =\frac{1}{2}mr^{2}(1+j)(\omega_{1}^{2}+\omega_{3}^{2})+mgr\;\sin\;\beta . & \end{array}$$

From energy conservation, at the rim $\left(\beta =\frac{\pi }{2}\right)$, $E_{\text{rim}}=\frac{1}{2}m(1+j)v^{2}+mgr$, using equation (3.19). Hence, from equation (4.7), we have


(4.8) $$\begin{array}{lcr} & \omega_{1}^{2}+\omega_{3}^{2}=\frac{v^{2}}{r^{2}}+\frac{2g}{r(1+j)}(1-\sin\;\beta ). & \end{array}$$

From equation (3.47), we have $\omega_{1}=\dot{\beta }$. To find $\omega_{3}$, we divide equation (3.45) by equation (3.47) to find


(4.9) $$\begin{array}{lcr} & \frac{d\omega_{3}}{d\beta }=-\frac{r\omega_{3}\sin\;\beta }{R-r\;\cos\;\beta }. & \end{array}$$

On integrating equation (4.9), using equation (3.20), we find^13^


(4.10) $$\begin{array}{lcr} & \omega_{3}(\beta )=-\frac{v\delta }{r(R-r\;\cos\;\beta )}. & \end{array}$$

Hence, from equation (4.7), we have


(4.11) $$\begin{array}{lcr} & {\dot{\beta }}^{2}=\frac{v^{2}}{r^{2}}\left(1-\frac{\delta^{2}}{(R-r\;\cos\;\beta {)}^{2}}\right)+\frac{2g}{r(1+j)}(1-\sin\;\beta ). & \end{array}$$

The elimination of $\omega_{3}$ from equation (4.8) reduces $\left(\beta ,\omega_{1},\omega_{3}\right)$-space to the plane $(\beta ,\omega_{1})\equiv \left(\beta ,\dot{\beta }\right)$*,* using equation (3.47). For each energy level, this is not a plane section but a curved surface determined by equation (4.10). Then equation (4.11) is the closed form expression for the $\left(\beta ,\dot{\beta }\right)$-phase-plane dynamics for the spin-free rim motion.^14^

By combining equations (4.2) and (4.10), evaluated at $\beta =\beta_{e}$, we find an implicit expression for $\beta_{e}$, given by


(4.12) $$g(R-r\cos\beta_{e}{)}^{3}=v^{2}\delta^{2}(1+j)\tan\beta_{e}.$$

**Figure 5.**
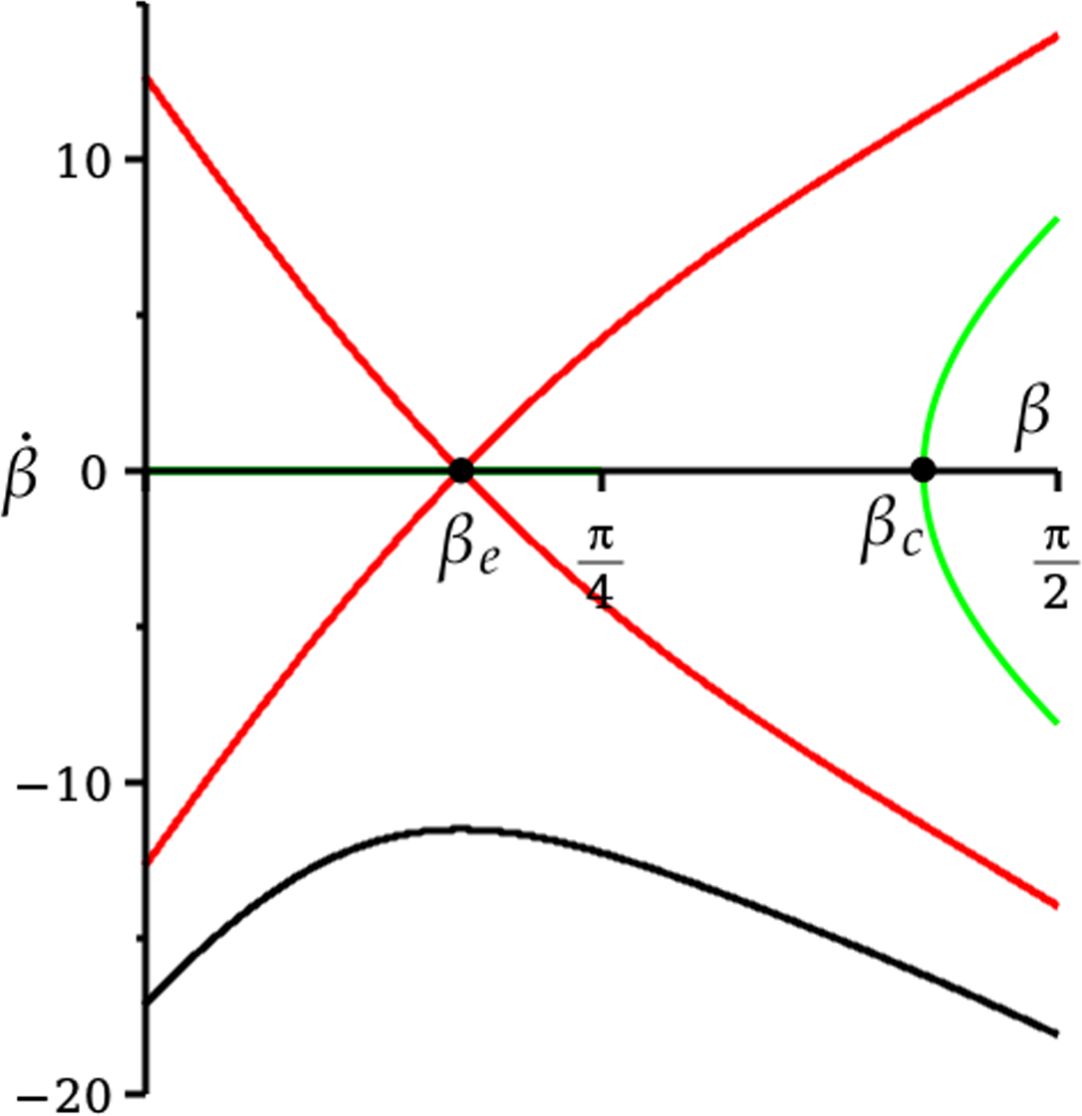
Phase-plane dynamics for spin-free rim motion, using equation (4.11). All trajectories have vδ=0.023, $\beta_{e}=0.54$. For the green trajectory, $\left(\frac{\delta }{R},v)=(0.93,0.46\right)$, $\beta_{c}=1.34$, and the golf ball undergoes a *rim lip out*. For the black trajectory, $\left(\frac{\delta }{R},v)=(0.74,0.575\right)$, and the golf ball goes into the hole. The red trajectories are separatrices, corresponding to $\left(\frac{\delta }{R},v)=(0.82,0.52\right)$.

In figure 5, we choose vδ=0.023, and hence $\beta_{e}=0.54$ from equation (4.12). Then we plot three trajectories in the $\left(\beta ,\dot{\beta }\right)$-phase plane^15^ with the same value of $\beta_{e}$. The green and black trajectories start at the rim with $\beta =\frac{\pi }{2}$, and with $\dot{\beta }<0$ given by equation (3.20), since $\dot{\beta }=\omega_{1}$ from equation (3.47).

For the green trajectory, δ=0.05 and so $(\frac{\delta }{R},v)=\left(0.93,0.46\right)$. The golf ball undergoes a *rim lip out*; the centre of mass tips into the hole, reaching a minimum value of $\beta =\beta_{c}=1.34$, but then pops back out again, to leave the rim with the same values of $\omega_{1,3}$ given by equation (3.20) as it entered. For the black trajectory, δ=0.04 and so $(\frac{\delta }{R},v)=\left(0.74,0.575\right)$, and the golf ball goes into the hole. The red trajectories are separatrices of the dynamics, corresponding to δ=0.04423, hence $(\frac{\delta }{R},v)=\left(0.82,0.52\right)$.

We will need to find how the angle α changes during lip outs. By combining equations (3.46) and (4.10) and then dividing by equation (3.47), we find


(4.13) $$\begin{array}{lcr} & \frac{d\alpha }{d\beta }=\frac{v\delta }{\left(R-r\;\cos\;\beta {)}^{2}\omega_{1}(\beta \right)}. & \end{array}$$

Hence, in the rim lip out, the trajectory undergoes an angle of turn (or ‘roll-around angle’ [4]) given by


(4.14) $$\Delta \alpha =2v\delta \int_{\frac{\pi }{2}}^{\beta_{c}}\frac{d\beta }{\left(R-r\cos\beta {)}^{2}\omega_{1}(\beta \right)},$$

where $\omega_{1}(\beta_{c})=0$ (figure 5). For the green trajectory in figure 5, we find $\Delta \alpha =1.058=60.6^{\circ }$. Choosing $\left(\frac{\delta }{R},v\right)$ closer to the red trajectory will give arbitrarily large values of Δα.

Finally, from equation (3.48), we find


(4.15) $$F_{2}(\beta )=\frac{mg}{\left(1+j\right)}[(j+3)\sin\beta -2]-\frac{mv^{2}}{r}\left(1-\frac{R\delta^{2}}{(R-r\cos\beta {)}^{3}}\right),$$

which must be positive for contact to be maintained between golf ball and rim in spin-free rim motion.

**Figure 6.**
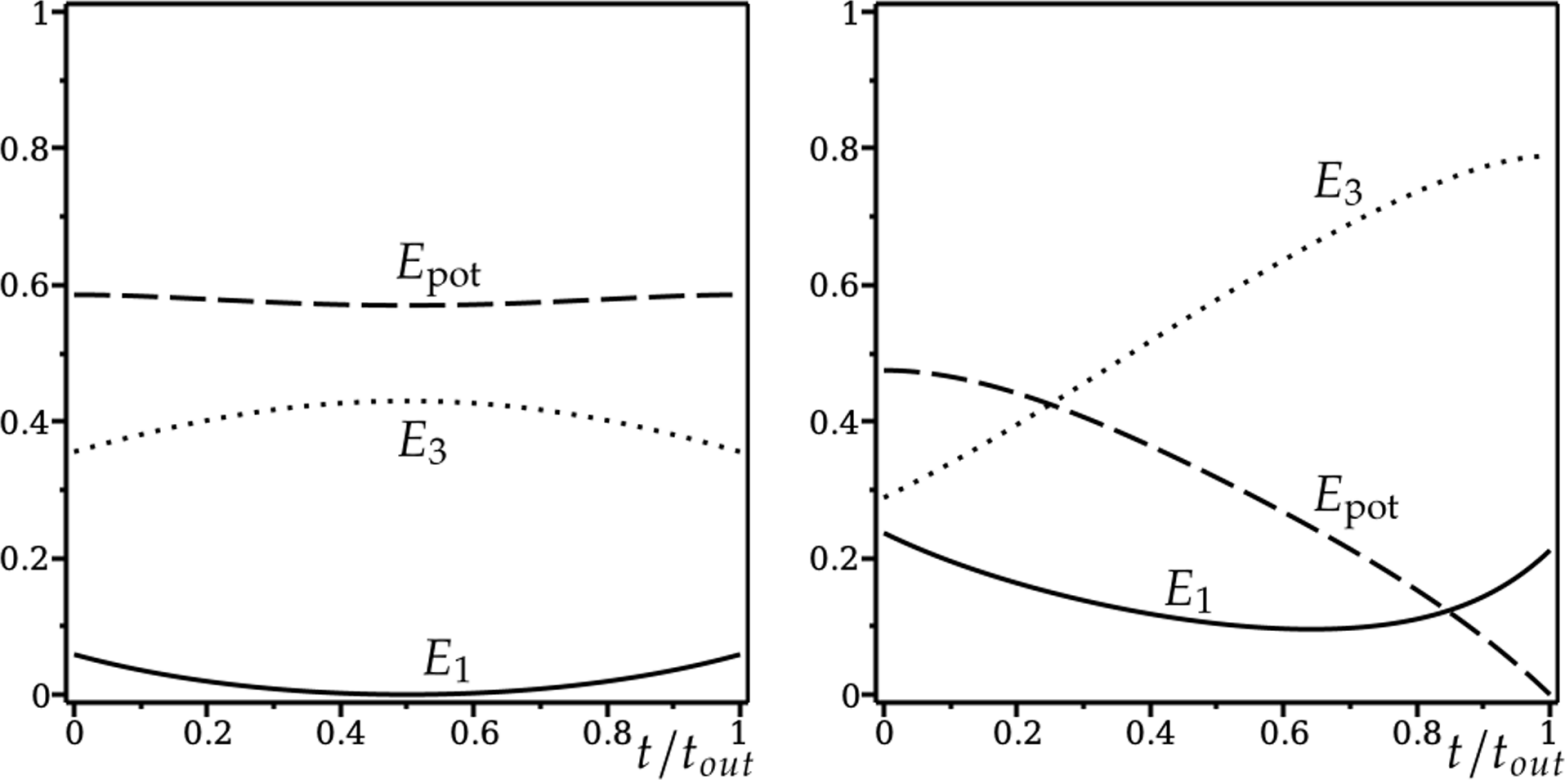
Relative energies $E_{1,3,\text{pot}}$ defined in equation (3.55) along (a) the green (lip out) and (b) the black (into hole) trajectories in figure 5, plotted as a function of $t/t_{out}$, where $t_{out}$ is the time taken to leave the rim. Solid line: $E_{1}$. Dotted line: $E_{3}$. Dashed line: $E_{\text{pot}}$.

We can understand the difference between the green and black trajectories in figure 5, as follows. In figure 6, we plot $E_{1,3,\text{pot}}$ from equation (3.55) against $t/t_{out}$ for both trajectories ($E_{2}\equiv 0$), where $t_{out}$ is the time taken to leave the rim, either by returning to the green or by entering the hole.

In figure 6a, the initial relative energy due to pitching $E_{1}$ (solid line) is a fraction of the initial relative energy due to rolling $E_{3}$ (dotted line). As the motion progresses, there is a small exchange between $E_{1,3,\text{pot}}$ that is restored in the second half of the motion, after pitching is reversed. The angular momentum due to pitching (into the hole) is not enough to overcome the angular momentum due to rolling (around the rim), and the golf ball lips out, without entering the hole. Note that the dry friction torque $M_{2}^{\text{rim}}$ that maintains spin-free rim motion (not shown) varies from −0.03 to 0.03 during this rim lip out.

In figure 6b, with different initial conditions for $\omega_{1,3}$, $E_{1}$ is comparable to $E_{3}$, initially. As the motion progresses, pitching is not reversed. Instead, $E_{\text{pot}}$ tends to zero, with a corresponding increase in $E_{3}$ as the golf ball enters the hole. The angular momentum due to pitching (into the hole) is enough for the golf ball to enter the hole, even though the angular momentum due to rolling (around the rim) increases significantly. In this case, the dry friction torque $M_{2}^{\text{rim}}$ varies from −0.07 to −0.17 (not shown).

### 4.3. Regions in the $\left(\frac{\delta }{R},v\right)$-plane for spin-free rim motion

In this section, we use results from the previous section to find analytic expressions for boundaries, within the $\left(\frac{\delta }{R},v\right)$-plane, which separate the different types of behaviour of the golf ball in spin-free rim motion (see [8, fig. 12] for numerical results).

#### 4.3.1. Initial fall from the rim

For sufficiently large v, Holmes [8] showed that the golf ball separates from the rim on arrival. There is no rim motion, and the golf ball subsequently behaves like a projectile. From equation (4.15), $F_{2}(\frac{\pi }{2})=0$ when


(4.16) $$\begin{array}{lcr} & 0=mg-\frac{mv^{2}}{r}\left(1-\frac{\delta^{2}}{R^{2}}\right), & \end{array}$$

which can be rewritten^16^


(4.17) $$\begin{array}{lcr} & v=c_{1}(\delta )=\sqrt{\frac{gr}{1-\delta^{2}/R^{2}}}. & \end{array}$$

**Figure 7.**
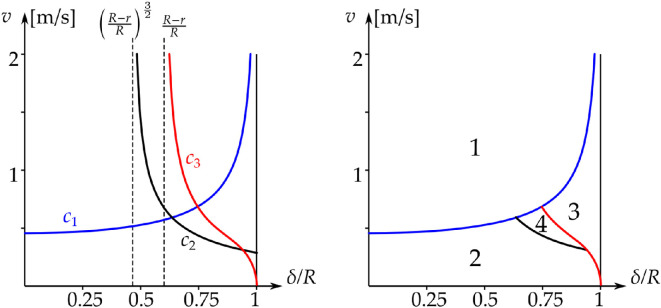
Analytical boundaries of the different behaviours on the $\left(\frac{\delta }{R},v\right)$-plane. Left panel: the boundary curves $c_{1,2,3}$ and their vertical asymptotes. Right panel: the regions of the different behaviour. 1: Fall from the rim at the beginning of the rim motion $\left(\beta =\frac{\pi }{2}\right)$. 2: Fall during rim motion with $\beta \in (0,\frac{\pi }{2})$. 3: Rim lip out, without reaching the hole motion. 4: Trajectories reach the hole motion (β=0) and fall into hole.

Equation (4.17), originally obtained by Holmes [8, eq. 6], is shown in blue in figure 7. In region 1, the golf ball separates from the rim, and the rim equations no longer apply.^17^ Below the blue curve $v=c_{1}(\delta )$, the golf ball can start spin-free rim motion.

#### 9.3.2. Fall during rim motion

It is possible that the golf ball can fall into the hole during rim motion, for some $\beta \in \left(0,\frac{\pi }{2}\right)$, by losing contact with the rim. Consider the time derivative of the normal force $F_{2}$ at the beginning of the rim motion. We find


(4.18) $$\begin{array}{lcr} & {\frac{dF_{2}}{dt}|}_{\beta =\pi /2}=\dot{\beta }{\frac{dF_{2}}{d\beta }|}_{\beta =\pi /2}=\frac{-3\omega_{1}mv^{2}\delta^{2}}{R^{3}}>0, & \end{array}$$

because $\omega_{1}$ is initially negative (see equation (3.20)). Hence, if $F_{2}$ is positive at the beginning of the rim motion, it starts increasing. However, $F_{2}$ can later decrease, and the golf ball can fall from the rim when $F_{2}=0$. $F_{2}$ vanishes exactly at the end of the rim motion when β=0. From equation (4.15), $F_{2}(0)=0$ when


(4.19) $$\begin{array}{lcr} & 0=-\frac{2mg}{1+j}-\frac{mv^{2}}{r}\left(1-\frac{R\delta^{2}}{(R-r{)}^{3}}\right), & \end{array}$$

which becomes


(4.20) $$\begin{array}{lcr} & v=c_{2}(\delta )=\sqrt{\frac{2gr/(1+j)}{R\delta^{2}/(R-r{)}^{3}-1}}. & \end{array}$$

The result is shown in black in figure 7. The curve $v=c_{2}(\delta )$ can only exist below region 1. Below $v=c_{2}(\delta )$ itself, the ball separates from the rim during the rim motion and falls into the hole. Region 2 of figure 7 corresponds to region CF1 of [8, fig. 12], where the curve $v=c_{2}(\delta )$ was calculated numerically.

Note that curves $v=c_{1,2}(\delta )$ are projections of the surface $F_{2}(\beta )=0$ in equation (4.15) onto the $\left(\frac{\delta }{R},v\right)$-plane. Hence, in region 2, the value of β when $F_{2}=0$ varies from $\beta =\frac{\pi }{2}$ at $\frac{\delta }{R}=0$ to β=0 when $v=c_{2}(\delta )$.

#### 4.3.3. Separatrices

The separatrices in figure 5 of the one-parametric family of saddles form a two-dimensional surface in the three-dimensional phase space. We search for the initial conditions in the $\left(\frac{\delta }{R},v\right)$-plane that lead to one of these saddles: these satisfy the conditions $\dot{\beta }(\beta_{e})=\ddot{\beta }(\beta_{e})=0$. From equation (4.11), we find


(4.21)β˙2(βe)=v2r2(1−δ2(R−rcos⁡βe)2)+2gr(1+j)(1−sin⁡βe)=0,(4.22)β¨(βe)=−gcos⁡βer(1+j)+v2δ2sin⁡βer(R−rcos⁡βe)3=0.

We cannot eliminate $\beta_{e}$ from equations (4.21) and (4.22), so the curve cannot be expressed analytically in the form $v=c_{3}(\delta )$. Instead, we use $\beta_{e}$ to obtain a parametric form of the curve


(4.23)v2(βe)=g(1+j)sin⁡βe(cos⁡βe(R−rcos⁡βe)−2rsin⁡βe(1−sin⁡βe)),(4.24)δ2(βe)=cos⁡βe(R−rcos⁡βe)3(cos⁡βe(R−rcos⁡βe)−2rsin⁡βe(1−sin⁡βe)).

**Figure 8.**
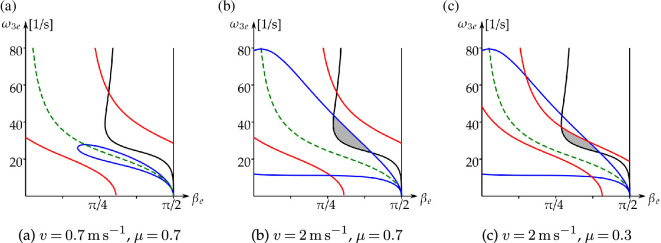
Limiting condition boundaries of non-trivial steady-state rim solutions with spin equations (5.2) and (5.3). Black: stability loss $h(\beta_{e},\omega_{3e})=0$ equation (5.6). Red: slipping and separation equation (5.7). Blue: kinetic energy equation (5.9). The green dashed line corresponds to $\omega_{2e}(\beta_{e},\omega_{3e})=0$ in equation (5.3). The steady state satisfies all the criteria in the grey areas.

**Figure 9.**
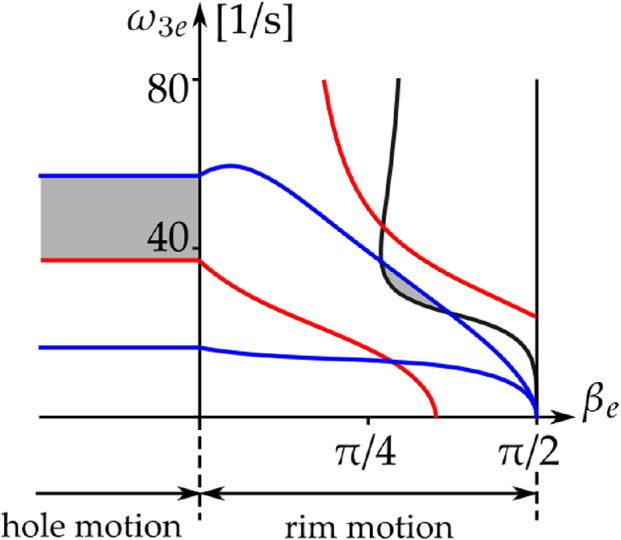
Limiting conditions of the steady rim *and* hole solutions equations (5.2) and (5.3) and equations (5.10) and (5.11) in the $\left(\beta_{e},\omega_{3e}\right)$-plane, for μ=0.5, v=1.5 m s^−1^. The steady states satisfy all the relevant criteria in the grey areas.

The result is shown in red in figure 7. The curve $v=c_{3}(\delta )$ can only exist below region 1. With initial conditions in the $\left(\frac{\delta }{R},v\right)$-plane, trajectories on $v=c_{3}(\delta )$ are separatrices of the saddles. The red separatrices in figure 5 correspond to values of $\left(\frac{\delta }{R},v\right)$-plane on $v=c_{3}(\delta )$. The green trajectory (rim lip out) in figure 5 corresponds to values of $\left(\frac{\delta }{R},v\right)$-plane in region 3; the black trajectory in figure 5 corresponds to values of $\left(\frac{\delta }{R},v\right)$-plane in region 4. We can analytically calculate the intersections of the curves $c_{1,2,3}(\delta )$. We find $P_{12}\equiv \left(\frac{\delta }{R},v{)}_{12}\approx (0.639,0.594\right)$, $P_{13}\equiv \left(\frac{\delta }{R},v{)}_{13}\approx (0.748,0.689\right)$ and $P_{23}\equiv \left(\frac{\delta }{R},v{)}_{23}\approx (0.940,0.316\right)$. These values are close to the values in [4, figs. 8, 9], which were computed from numerical simulations.^18^

The value of $\beta_{e}$ decreases as v increases along $v=c_{3}(\delta )$. But the fact that $c_{3}(\delta )$ cannot exist in region 1 gives us another bound on $\beta_{e}$. At $P_{13}$, we have vδ=0.0278, corresponding to $\beta_{e}=0.3376\approx 19.34^{\circ }$ from equation (4.12).

### 4.4. Spin-free hole motion

In region 4 of figure 7, the golf ball remains in contact with the rim until β=0, at which point the golf ball enters the hole. Let us assume that the spin-free equations of hole motion equations (3.49)–(3.52) apply. Steady-state spin-free hole motion is not possible.^19^ But these equations can be integrated exactly (note we reset time so that t=0 when the hole motion begins at β=0).

From equations (3.49) and (3.50), we find $\omega_{1}(t)=-\frac{gt}{r(1+j)}+\omega_{1}(0)$, $\omega_{3}(t)=\omega_{3}(0)$, where $\omega_{1,3}(0)<0$ are the values of $\omega_{1,3}$ as the golf ball enters the hole.^20^ From equation (3.52), we find


(4.25) $$\begin{array}{lcr} & \frac{1+\sin\;\beta }{\cos\;\beta }=\exp\left(-\frac{gt^{2}}{r(1+j)}+\omega_{1}(0)t\right). & \end{array}$$

Hence, $\beta \to -\frac{\pi }{2}$ as t→∞ and the golf ball falls to the bottom of the hole in region 4.

Note that


(4.26) $$M_{2}^{\text{\{hole\}}}=\frac{mgr^{3}\omega_{3}(0)}{R-r}\left(\frac{gt}{r(1+j)}+|\omega_{1}(0)|\right),$$

and so $M_{2}^{\text{hole}}$ has to increase linearly with time in order to maintain spin-free hole motion.

### 4.5. Spin-free motion: summary

Overall, care should be taken in the interpretation of figure 5. It is a projection onto the $\left(\beta ,\dot{\beta }\right)$-phase plane of dynamics in $(\beta ,\dot{\beta },\omega_{3})\equiv \left(\beta ,\omega_{1},\omega_{3}\right)$-phase space. To obtain the full picture, we need to vary vδ so that we always cross the boundary $v=c_{3}(\delta )$ between intersections $P_{13}$ and $P_{23}$. Hence, we take vδ∈[0.0160,0.0278]. The position of the saddle at $\beta =\beta_{e}$ will then vary according to equation (4.12). In figure 7, the curves $c_{1,2,3}(\delta )$ separate the $\left(\frac{\delta }{R},v\right)$-plane into four regions:

— *Region 1*. Fall from the rim at the beginning of the rim motion (β=π/2).
— *Region 2*. Fall during rim motion with β∈(0,π/2).
— *Region 3*. Rim lip out.
— *Region 4*. Trajectories reach the hole motion (β=0), and the golf ball falls into the hole.

For δ/R≪1, the path of the ball almost goes through the centre of the hole, and $|\omega_{3}|\ll \left|\omega_{1}\right|$. In this case, we get the behaviour in region 1 of figure 7. In region 2, $\left|\omega_{3}\right|$ and $\left|\omega_{1}\right|$ are comparable, but the pitch $\omega_{1}$ into the hole dominates. In region 4, pitch still governs the motion of the golf ball, but rolling becomes more prominent. Finally, in region 3, for δ→R, the path of the ball is far from the centre, and $|\omega_{3}|\gg \left|\omega_{1}\right|$. Pitch has little effect, and the golf ball rolls around the rim and away from the hole, in a rim lip out.

## 5. Steady-state motion with spin

In this section, we assume that the spin angular velocity $\omega_{2}(t)\neq 0$ in general, and we dispense with the dry friction torques $M_{2}^{\text{rim, hole}}$. The governing equations for the rim motion with spin are equations (3.28)–(3.32), and those of the hole motion with spin are equations (3.34)–(3.38).

We look for steady-state solutions (equilibria) of the (rim, hole) equations of motion and consider their stability. Since the α motion is cyclic, we consider only the four-dimensional dynamics of $x(t)\equiv (\beta ,\omega_{1},\omega_{2},\omega_{3}{)}^{T}$.

### 5.1. Steady-state rim motion with spin

The rim motion is similar to the basketball motion [19], in the case of pure rolling. The main difference is that the golf ball must always arrive at the rim with $\beta =\frac{\pi }{2}$. There are two steady-state solutions of equations (3.28) to (3.30) and (3.32) (see also [19, §3.1]).

#### 5.1.1. Trivial rim solution with spin

The solution


(5.1) $$\begin{array}{lcr} & x_{er1}=(\frac{\pi }{2},0,\omega_{2e},0{)}^{T} & \end{array}$$

for constant $\omega_{2e}$ is the trivial steady state of the rim motion. It corresponds to the golf ball spinning about the vertical axis, at a fixed point of the rim, with arbitrary fixed angular velocity $\omega_{2e}$. It is unstable [19]. This solution was also found by [8], who pointed out that it is inconsistent with the golf ball arriving at the hole rim with non-zero translational velocity.

#### 5.1.2. Non-trivial rim solution with spin

The solution


(5.2) $$\begin{array}{lcr} & x_{er2}=(\beta_{e},0,\omega_{2e}(\beta_{e},\omega_{3e}),\omega_{3e}{)}^{T} & \end{array}$$

for constant $\beta_{e}$ and $\omega_{3e}$ is the non-trivial steady state of the rim motion with spin, where


(5.3) $$\begin{array}{lcr} & \omega_{2e}(\beta_{e},\omega_{3e})=\frac{\left(1+j\right)}{j}\omega_{3e}\tan\;\beta_{e}-\frac{g}{jr^{2}\omega_{3e}}(R-r\;\cos\;\beta_{e}). & \end{array}$$

Solving equation (5.3) for $\omega_{3e}$, we find


(5.4) $$\begin{array}{lcr} & \omega_{3e}=\frac{j}{2(1+j)}\omega_{2e}\cot\;\beta_{e}\pm \sqrt{\frac{j^{2}}{4(1+j{)}^{2}}\omega_{2e}^{2}\cot^{2}\;\beta_{e}+\frac{g}{r^{2}(1+j)}(R-r\;\cos\;\beta_{e})\;\cot\;\beta_{e}}. & \end{array}$$

Equation (5.4) reduces to equation (4.2) when $\omega_{2e}=0$. The steady-state equation (5.2) corresponds to the golf ball rolling around the rim of the hole with zero pitch and arbitrary angular velocity $\omega_{3e}$ in the $g_{3}$ direction, while spinning with angular velocity $\omega_{2e}(\beta_{e},\omega_{3e})$ about the $g_{2}$-axis, which itself is inclined at an angle $\beta_{e}$ to the horizontal (figure 4). The steady-state equations (5.2) and (5.3) form a two-parametric surface in the four-dimensional state space of the rim equations with spin.^21^

#### 5.1.3. Limiting conditions of the non-trivial rim solution with spin

*Stability loss.* The linear stability of the non-trivial rim steady-state $x_{er2}$ in equation (5.2) is governed by the eigenvalues $\lambda =\lambda_{i},\;\left(i=1..4\right)$ of the characteristic equation


(5.5) $$\lambda^{2}(\lambda^{2}-h(\beta_{e},\omega_{3e}))=0,$$

where^22^


(5.6) $$\begin{array}{lcr} & h(\beta_{e},\omega_{3e})=\frac{r\omega_{3e}^{2}[R\;\cos\;\beta_{e}-(1+j)r]}{(1+j)(R-r\;\cos\;\beta_{e}{)}^{2}}-\frac{g\;\sin\;2\beta_{e}}{\left(1+j)(R-r\;\cos\;\beta_{e}\right)}+\frac{g^{2}\cos\;\beta_{e}(R-r\;\cos\;\beta_{e})}{r^{3}(1+j{)}^{2}\omega_{3e}^{2}}. & \end{array}$$

From equation (5.5), two of the eigenvalues are zero. As in [19], if $h(\beta_{e},\omega_{3e})>0$, then the other two eigenvalues are real, and the non-trivial rim steady-state $x_{er2}$ in equation (5.2) is a degenerate saddle. If $h(\beta_{e},\omega_{3e})<0$, then it is a degenerate centre.^23^

In [4, p. 94], numerical calculations were presented, which led the authors to believe that ‘… the linearized eigenstructure [of the rim motion] has the same character about all equilibrium solutions; namely two eigenvalues at the origin and two real roots of equal magnitude but opposite sign’. That corresponds to $h(\beta_{e},\omega_{3e})>0$. But it is possible that $h(\beta_{e},\omega_{3e})<0$; see the grey areas in figure 8, where the curve $h(\beta_{e},\omega_{3e})=0$ is shown in black (note that $h(\beta_{e},\omega_{3e})>0$ to the left of the curve, and $h(\beta_{e},\omega_{3e})<0$ to the right of it). These degenerate centres cannot be reached when starting from $\beta =\frac{\pi }{2}$. We discuss the properties of the vertical tangent to the curve $h(\beta_{e},\omega_{3e})=0$ and its asymptote in appendix E.

*Slipping and separation.* By substituting equations (5.2) and (5.3) into equation (3.33), the dynamic condition (equation (3.27)) to ensure rolling without slipping becomes


(5.7) $$\begin{array}{lcr} & \left|g\;\cos\;\beta_{e}-\frac{r^{2}\omega_{3e}^{2}\sin\;\beta_{e}}{R-r\;\cos\;\beta_{e}}\right|\leq \mu \left(g\;\sin\;\beta_{e}+\frac{r^{2}\omega_{3e}^{2}\cos\;\beta_{e}}{R-r\;\cos\;\beta_{e}}\right). & \end{array}$$

In figure 8, this condition is satisfied at all points between the red curves. Note that in the limit case μ→∞, equation (5.7) leads to the condition of steady motion without separation, which is always satisfied^24^ for the region $\beta_{e}\in \left[0,\pi /2\right]$ (the right-hand side of equation (5.7) cannot be negative in this region).

*Kinetic energy.* The golf ball arrives at the rim of the hole with finite kinetic energy. But the expression for the non-trivial rim steady-state $\omega_{2e}(\beta_{e},\omega_{3e})$ equation (5.3) can diverge, when $\beta_{e}\to \pi /2$ or $\omega_{3e}\to 0$. There must be some constraint on the steady-state angular velocities, based on the arrival kinetic energy. The golf ball’s kinetic energy T must satisfy


(5.8) $$\begin{array}{lcr} & T=\frac{1}{2}m|v_{G}{|}^{2}+\frac{1}{2}jmr^{2}|\omega {|}^{2}\leq \frac{1}{2}mv^{2}. & \end{array}$$

By substituting equations (3.11), (5.2) and (5.3) into equation (5.8), we obtain


(5.9) $$\begin{array}{lcr} & jr^{2}\omega_{2e}^{2}(\beta_{e},\omega_{3e})+(1+j)r^{2}\omega_{3e}^{2}\leq v^{2}. & \end{array}$$

In figure 8, this condition is satisfied at all points between the blue curves.

### 5.2. Steady hole motion with spin

Hole motion with spin has been considered by several authors [5,6,12–14]. There is one steady-state solution of equations (3.34) –(3.36) and (3.38), given by


(5.10) $$x_{eh}=(\beta_{e},0,\omega_{2e}(\omega_{3e}),\omega_{3e}{)}^{T},$$

where


(5.11) $$\begin{array}{lcr} & \omega_{2e}(\omega_{3e})=-\frac{g(R-r)}{jr^{2}\omega_{3e}}. & \end{array}$$

This motion corresponds to the golf ball rolling inside the hole, at an arbitrary depth (determined by the choice of $\beta_{e}$), with an arbitrary angular velocity $\omega_{3e}$, while spinning about the $g_{2}$ axis with angular velocity $\omega_{2e}(\omega_{3e})$.

#### 5.2.1. Limiting conditions

*Stability.* The eigenvalues of the steady-state $x_{eh}$ equations (5.10) and (5.11) are given by


(5.12) $$\begin{array}{lcr} & \lambda =0,0,\pm i\frac{r\omega_{3e}}{\left(R-r\right)}\sqrt{\frac{j}{1+j}}, & \end{array}$$

corresponding to a degenerate centre, and so $x_{eh}$ is (neutrally) stable. A general perturbation results in oscillations around $x_{eh}$ of frequency $\frac{r\omega_{3e}}{\left(R-r\right)}\sqrt{\frac{j}{1+j}}$.

*Slipping and separation.* The dynamic condition for rolling without slipping is obtained by substituting equation (5.11) into equation (3.27), to give


(5.13) $$\frac{g(R-r)}{r^{2}}\leq \mu \omega_{3e}^{2}.$$

Consequently, a minimum rolling angular velocity $\omega_{3e}$ is needed to require a sufficient amount of normal force [14]. The condition of separation is the limit μ→∞ at equation (5.13), which leads to the trivial result $\omega_{3e}^{2}\geq 0$.

*Kinetic energy.* The limitation imposed on the hole motion by the kinetic energy of the putt is calculated in a similar way to that of the rim motion. Let us assume that the golf ball arrives at the rim of the hole with speed v and enters the hole directly. Then we substitute equations (3.11), (5.10) and (5.11) into equation (5.8), to find


(5.14) $$\begin{array}{lcr} & \frac{g^{2}(R-r{)}^{2}}{jr^{2}\omega_{3e}^{2}}+(1+j)r^{2}\omega_{3e}^{2}\leq v^{2}. & \end{array}$$

The left-hand side of equation (5.14) has a minimum energy level when $v=\hat{v}$ at $\omega_{3e}={\hat{\omega }}_{3e}$, where


(5.15) $$\begin{array}{lrr} & {\hat{\omega }}_{3e}^{2}=\frac{g(R-r)}{r^{2}\sqrt{j(1+j)}},\;{\hat{v}}^{2}=2g(R-r)\sqrt{\frac{1+j}{j}}. & \end{array}$$

For the golf ball and hole, ${\hat{\omega }}_{3e}=30.64,\;\hat{v}=1.09$.

In figure 9, we show the limiting conditions for the rim and hole solutions next to one another, when μ=0.5, v=1.5 m s^−1^. The slip and the energy boundaries are continuously connected.

## 6. Motion with spin

In this section, we consider the time-dependent equations of rim motion with spin equations (3.28)–(3.33) and of hole motion with spin equations (3.34)–(3.39). In both cases, we can find analytic solutions to the problem. But for the rim problem, these solutions are unwieldy, and we revert to numerical calculations.

### 6.1. Rim motion with spin

We consider equations (3.28)–(3.33). If we divide equations (3.29) and (3.30) by equation (3.32) and then eliminate $\omega_{2}$, we find


(6.1) $$\begin{array}{lcr} & \frac{d^{2}\omega_{3}}{d\beta^{2}}+\frac{r\;\sin\;\beta }{\left(R-r\;\cos\;\beta \right)}\frac{d\omega_{3}}{d\beta }+\frac{\left[jR^{2}-(1+j)r^{2}+rR\;\cos\;\beta \right]}{(1+j)(R-r\;\cos\;\beta {)}^{2}}\omega_{3}=0. & \end{array}$$

With suitable conditions on $\omega_{3},\;\frac{d\omega_{3}}{d\beta }$ at $\beta =\frac{\pi }{2}$, this linear equation can be solved for $\omega_{3}(\beta )$ in terms of general Heun functions and their derivatives [23], which can then be used to find $\omega_{1,2}(\beta )$. This approach becomes unwieldy quickly. Instead, we perform the calculations numerically.

In [4, fig. 7], the authors numerically computed the boundary between regions 3 and 4 of figure 7 for three different sets of equations: (i) spin-free rim motion, (ii) rim motion with initial spin set to zero with no dry friction (pivoting) torque, and (iii) rim motion with initial spin set to zero with a small torque proportional to $F_{2}$. They showed that the boundary hardly changes between the three cases. But what about the solutions on either side of it?

#### 6.1.1. Zero initial spin

We ignore any torque. Then let us assume that the golf ball arrives at the rim with no-spin; hence, $\omega_{2}(\beta =\frac{\pi }{2})=0$. Then, we can still use the same initial conditions equation (3.20) for $\omega_{1,3}$ as we did for the spin-free case. But instead of spin remaining zero, spin may develop during the rim motion.

We compare our results with those of [4, figs. 4, 5]. In [4, fig. 4], the initial conditions of the golf ball at the rim were taken^25^ to be $(\frac{\delta }{R},v)=\left(0.89,0.60\right)$, which is in region 3 of figure 7 and in [4, fig. 5] $(\frac{\delta }{R},v)=\left(0.89,0.405\right)$, as well as in region 3. In both these cases, the golf ball lips out, with a far larger angle of turn in the second case (see [4, fig. 6]). We find excellent agreement, except that in [4, fig. 5], the time taken to lip out is smaller (0.75 s as opposed to 0.95 s).

**Figure 10.**
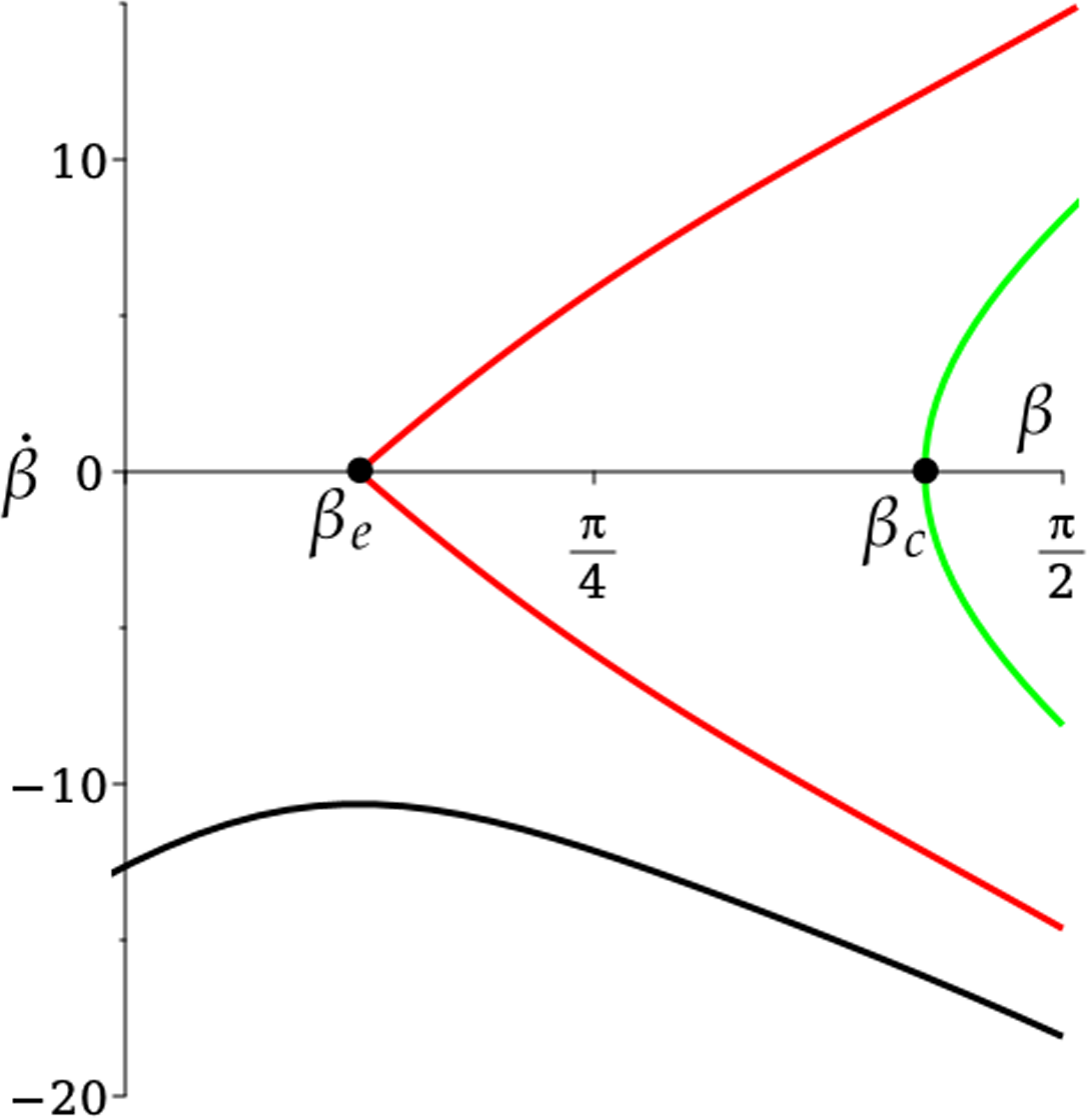
Phase-plane dynamics for rim motion with spin, computed using equations (3.28)–(3.32) with zero initial spin, where we include the same values of $\beta_{e,c}$ as in figure 5 for comparison. All trajectories have vδ=0.023. For the green trajectory, $(\frac{\delta }{R},v)=\left(0.93,0.46\right)$, and the golf ball undergoes rim lip out. For the black trajectory, $(\frac{\delta }{R},v)=\left(0.74,0.575\right)$, and the golf ball goes into the hole. The red trajectory has $(\frac{\delta }{R},v)=\left(0.81,0.53\right)$. It is close to the separatrix of the saddle.

How is the saddle structure of the spin-free rim motion in figure 5 affected? In order to compare the results of the rim equations with spin (but with zero initial spin) with those in figure 5, we must take vδ=0.023 and use the same initial conditions (equation (3.20)). The results are shown in figure 10, where we have taken the same values of both $\beta_{e,c}$ from figure 5. For the green trajectory $(\frac{\delta }{R},v)=\left(0.93,0.46\right)$*,* the golf ball undergoes a rim lip out, as before. The trajectory and the point $\beta =\beta_{c}$, $\dot{\beta }=0$ appear almost identical to the same case in figure 5. For the black trajectory with $(\frac{\delta }{R},v)=\left(0.74,0.575\right)$*,* the golf ball goes into the hole, also as before, but the value of $\dot{\beta }{|}_{\beta =0}\equiv \omega_{1}{|}_{\beta =0}$ is reduced. The red trajectory has δ=0.04354; hence, $(\frac{\delta }{R},v)=\left(0.81,0.53\right)$*,* which differs from figure 5, where $(\frac{\delta }{R},v)=\left(0.82,0.52\right)$ shows that the boundary between regions 3 and 4 in figure 7 has moved slightly. It is a close approximation to separatrices of the saddle in this problem, which is displaced from $\beta =\beta_{e}$. As with figure 5, care should be taken in the interpretation of figure 10. It is a projection onto the $\left(\beta ,\dot{\beta }\right)$-phase plane of dynamics in $(\beta ,\dot{\beta },\omega_{2},\omega_{3})\equiv \left(\beta ,\omega_{1},\omega_{2},\omega_{3}\right)$-phase space. It is straightforward to check that $F_{2}>0$ for all three trajectories in figure 10.

**Figure 11.**
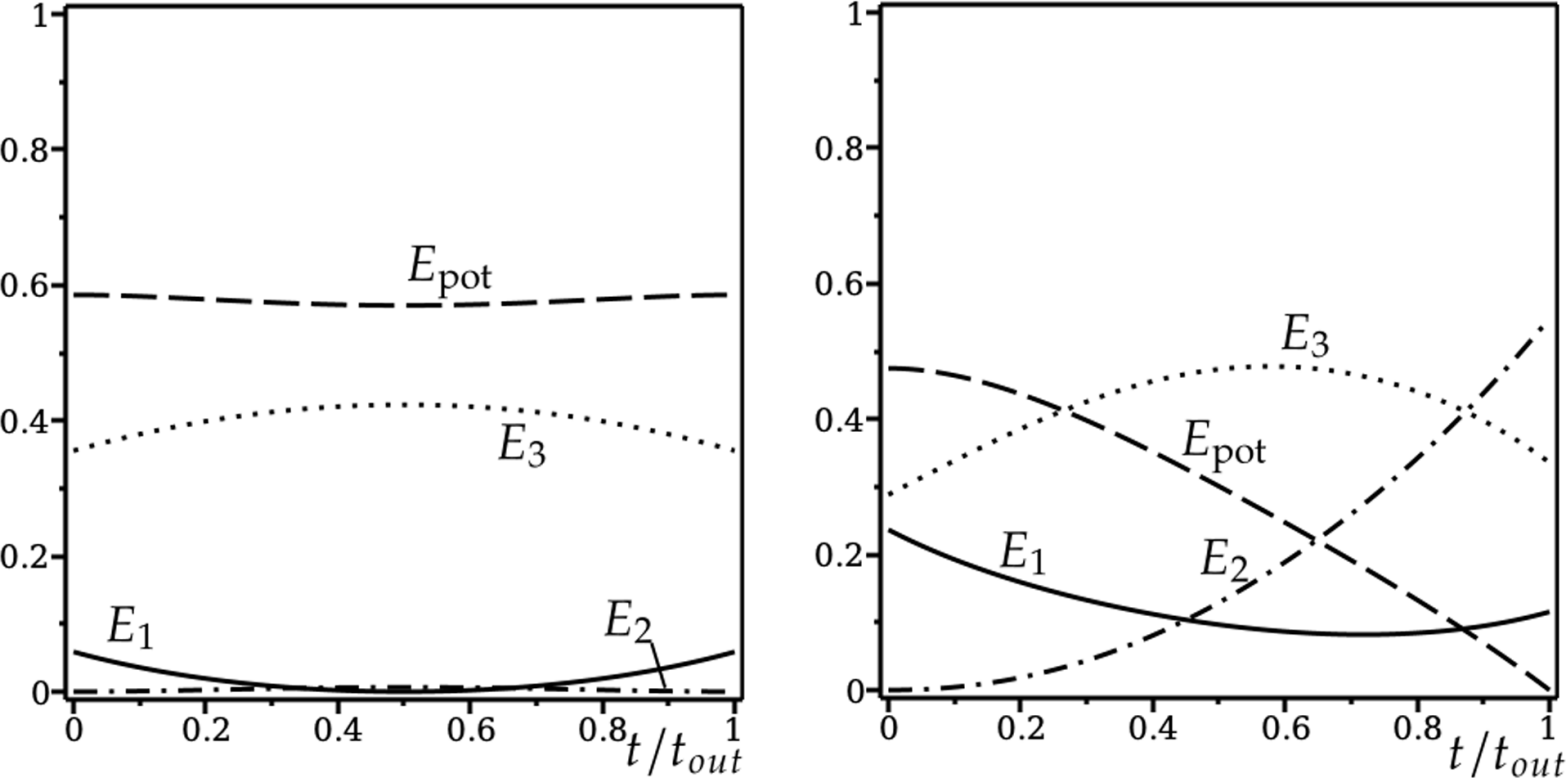
Relative energies $E_{1,2,3,\text{pot}}$ defined in equation (3.55) along (a) the green (lip out) and (b) the black (into hole) trajectories in figure 10 when $\omega_{2}(0)=0$, plotted as a function of $t/t_{out}$, where $t_{out}$ is the time taken to leave the rim. Solid line: $E_{1}$. Dash-dotted line: $E_{2}$. Dotted line: $E_{3}$. Dashed line: $E_{\text{pot}}$. Compare with spin-free rim motion in figure 6, where $E_{2}(t)\equiv 0$.

More insight can be gained by considering the relative energies $E_{1,2,3,\text{pot}}$ in equation (3.55). Results are shown in figure 11. We compare with the relative energies of the spin-free rim motion in figure 6, where $E_{2}(t)\equiv 0$, and the values of (δ,v) are the same.

For the green trajectory (left panel in both figures), there is almost no difference. Energy due to pitching $E_{1}$ is lost to energy due to rolling $E_{3}$, before being restored again as the golf ball returns to the rim. The potential energy $E_{pot}$ varies very little, and the spin energy $E_{2}$ only slightly increases from zero, decreasing to zero again as the golf ball leaves the rim. The rim lip out with zero initial spin is almost identical to the spin-free rim lip out. Very little spin is generated during the motion.

For the black trajectory (right panel in both figures), there are minor differences between the energy due to pitching $E_{1}$ and the potential energy $E_{pot}$. In figure 6b, the energy due to spin $E_{2}(t)\equiv 0$, and the energy due to rolling $E_{3}$ increases. But, in figure 11b, the spin energy $E_{2}$ rises inexorably, as the golf ball descends into the hole, at the expense of the energy due to rolling $E_{3}$, which itself is much smaller compared with the spin-free case.

From the rim lip out trajectory, it would appear that the spin-free equations are a fair approximation to the general equations with zero *initial* spin. But if the golf ball descends into the hole, those same spin-free equations give an inaccurate picture of the motion, which can have important implications for the subsequent hole motion (see below).

#### 6.1.2. Non-zero initial spin

In this case, we assume that the golf ball arrives at the rim with spin given by


(6.2) $$\begin{array}{lcr} & \omega_{2}(\beta =\frac{\pi }{2})=\varepsilon \frac{v}{r}. & \end{array}$$

We consider both positive (ε>0) and negative (ε<0) spin. In practice, we expect that |ε|≪1. The initial conditions (equation (3.20)) for $\omega_{1,3}$ remain the same, since $r^{2}(\omega_{1}^{2}+\omega_{3}^{2})=v^{2}$ from equation (3.11).

For the green (rim lip out) trajectory in figure 10, where $(\frac{\delta }{R},v)=\left(0.93,0.46\right)$ and ε=0, the relative energies $E_{1,2,3,\text{pot}}$ defined in equation (3.55) remain qualitatively the same for ε∈[−1,1]*,* and $F_{2}$ is always positive. The rim lip out still occurs.

For the black trajectory in figure 10, where $(\frac{\delta }{R},v)=\left(0.74,0.575\right)$ and ε=0, the golf ball enters the hole. The same is qualitatively true for ε∈(0,1]. For ε⪆0.3, we observe that $F_{2}<0$, and so the golf ball falls directly into the hole—no subsequent hole motion is possible. For ε<0, $F_{2}>0$ always. But for ε<−0.69, the golf ball experiences a rim lip out. We could explore this case in more detail, but even casual observation of putting indicates that |ε| would never become so large.

Hence, realistic values of non-zero initial spin (|ε|≪1) do not affect the behaviour of the golf ball when compared with zero initial spin on the rim.

For the spin-free case, once the golf ball entered the hole (region 4 of figure 7), it remained there (equation (4.25)). But we know [5–7,12–14] that a hole lip out from within a cylinder is possible in the presence of spin. We have seen that spin is generated on the rim, even when the golf ball arrives there with no-spin. Could the hole lip out be related to the spin of the golf ball as it enters the hole?

### 6.2. Hole motion with spin

Hole motion with spin is governed by equations (3.34)–(3.38). From equation (3.36), we see that $\omega_{3}(t)=\omega_{30}$ is a constant.^26^ There are two cases to consider: $\omega_{30}=0$ and $\omega_{30}\neq 0$.

#### 6.2.1. $\omega_{30}=0$

None of our simulations of the rim motion with spin end with $\omega_{3}=0$, and all of them end with the value of pitch $\omega_{1}<0$. Nevertheless, we can show, for initial values $\alpha (0)=\alpha_{0}$, $\omega_{1}(0)=\omega_{10}$, $\omega_{2}(0)=\omega_{20}$, that


(6.3) $$\begin{array}{lcr} & \alpha (t)=\alpha_{0},\;\omega_{1}(t)=\omega_{10}-\frac{gt}{r(1+j)},\;\omega_{2}(t)=\omega_{20},\;z(t)=r\left(\omega_{10}t-\frac{gt^{2}}{2r(1+j)}\right). & \end{array}$$

Since $\omega_{10}<0$, the golf ball eventually hits the bottom of the hole in this case. Note that z(t) is independent of the value of $\omega_{20}$, the value of spin at the end of the rim motion.

#### 6.2.2. $\omega_{30}\neq 0$

We can integrate equation (3.37) to give


(6.4) $$\begin{array}{lcr} & \alpha (t)=-\frac{r\omega_{30}}{\left(R-r\right)}t+\alpha_{0}. & \end{array}$$

By differentiating equation (3.35) with respect to time, and using equation (3.34), we find that $\omega_{2}(t)$ exhibits forced simple harmonic motion


(6.5) $$\begin{array}{lcr} & {\overset{"}{\omega }}_{2}+\Omega^{2}\omega_{2}=-\frac{g}{\left(1+j)(R-r\right)}\omega_{30}, & \end{array}$$

where the frequency Ω is given by^27^


(6.6) $$\begin{array}{lcr} & \Omega^{2}\equiv \frac{jr^{2}\omega_{30}^{2}}{(1+j)(R-r{)}^{2}}>0. & \end{array}$$

On integration of equation (6.5) and using equation (3.35), we find


(6.7) $$\begin{array}{lcr} & \omega_{2}(t)=\frac{r\omega_{10}\omega_{30}}{\Omega (R-r)}\sin\;\Omega t+\left(\omega_{20}+\frac{g(R-r)}{jr^{2}\omega_{30}}\right)\;\cos\;\Omega t-\frac{g(R-r)}{jr^{2}\omega_{30}}. & \end{array}$$

Then, using equation (3.35) again, we find


(6.8) $$\begin{array}{lcr} & \omega_{1}(t)=\omega_{10}\cos\;\Omega t-\frac{\Omega (R-r)}{r\omega_{30}}\left(\omega_{20}+\frac{g(R-r)}{jr^{2}\omega_{30}}\right)\;\sin\;\Omega t. & \end{array}$$

The vertical coordinate z of the centre of the golf ball G is given by z=rtan⁡β, from figure 3. Hence, from equation (3.38), we have


(6.9) $$\begin{array}{lcr} & \int \sec^{2}\;\beta d\beta =\int \omega_{1}dt. & \end{array}$$

From equations (6.8) and (6.9), we find^28^ that


(6.10) $$\begin{array}{lcr} & z(t)=\frac{r\omega_{10}}{\Omega }\sin\;\Omega t+\frac{\left(R-r\right)}{\omega_{30}}\left(\omega_{20}+\frac{g(R-r)}{jr^{2}\omega_{30}}\right)(\cos\;\Omega t-1). & \end{array}$$

Hence, the golf ball oscillates vertically in simple harmonic motion with frequency Ω as it goes around the hole (see also [5,6]).

It would appear from equation (6.10) that for $\omega_{30}\neq 0$, for some t>0, the golf ball must re-emerge from the hole.^29^ But the bottom of the hole is at z=−H. Hence, the golf ball of radius r will hit the bottom of the hole if z<−(H−r).

Set


(6.11) $$\begin{array}{lcr} & A=\frac{r\omega_{10}}{\Omega },\;B=\frac{\left(R-r\right)}{\omega_{30}}\left(\omega_{20}+\frac{g(R-r)}{jr^{2}\omega_{30}}\right). & \end{array}$$

Then, from equation (6.10), z(t)=Asin⁡Ωt+B(cos⁡Ωt−1)=γsin⁡(Ωt+ψ)−B, where A=γcos⁡ψ, B=γsin⁡ψ and $A^{2}+B^{2}=\gamma^{2}$, tan⁡ψ=B/A. We calculate $t_{min}>0$, the time when z(t) is a minimum value. It is straightforward to show that $\tan\;\Omega t_{min}=A/B$. If $z(t_{min})<-\left(H-r\right)$, the putt is successful (no hole lip out), which is equivalent to


(6.12) $$\begin{array}{lcr} & -\sqrt{A^{2}+B^{2}}-B+(H-r)<0, & \end{array}$$

which can be written


(6.13)F(ω10,ω20,ω30)≡A2−(H−r)[H−r−2B]>0,(6.14)=(1+j)(R−r)2ω102jω302+2(H−r)(R−r)ω30(ω20+g(R−r)jr2ω30)−(H−r)2>0.

Note that $\omega_{10}<0$, so the golf ball pitches into the hole at the end of the rim motion β=0, so that $\dot{z}{|}_{t=0}<0$ from equation (6.10) at the start of the hole motion. Since $\omega_{n0},\;n=1,2,3$ are functions of v,δ,ε, we can verify whether equation (6.13) is satisfied for the initial conditions of the golf ball when it arrives at the rim.

## 7. Combined rim and hole motion, with spin

In §4, for spin-free rim motion that reaches the hole (region 4 of figure 7), the golf ball always stays in the hole if the motion there is assumed to be spin-free as well. Hence, if there is to be a hole lip out, it must happen for hole motion with spin. The relevant governing equations (3.34)–(3.38) have a time-reverse symmetry with respect to $\omega_{1}=0$. So if $\omega_{1}=0$ is reached, a lip out will happen (provided the golf ball does not hit the bottom of the hole first).

**Figure 12.**
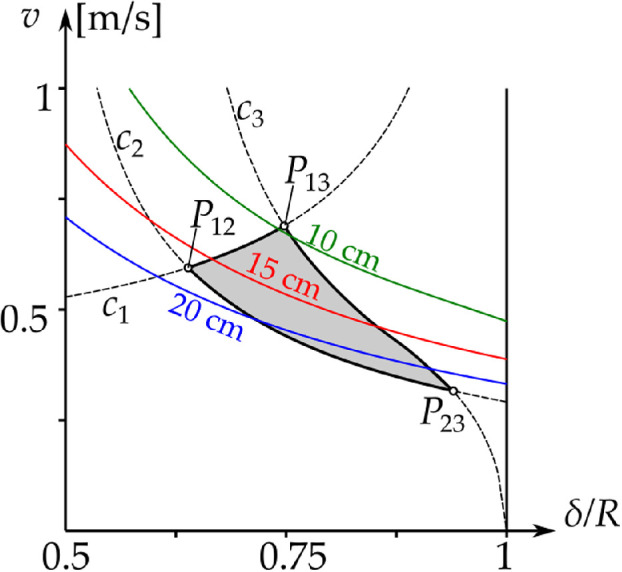
Region 4 of figure 7. The coloured lines are level sets of H(δ,v)=0 showing the minimum hole depth for the occurrence of a hole lip out for the given initial conditions. For the standard minimum hole depth H=0.102 m (see appendix A), a hole lip out can only occur in a tiny part of this region.

Let us assume spin-free rim motion, but allow for hole motion with spin. Then we can use the exact results from §4 in equation (6.13) to see if a hole lip out is possible. We have that $\omega_{20}\equiv 0$, and from equations (4.10) and (4.11) evaluated at β=0, we find


(7.1) $$\begin{array}{lcr} & \omega_{10}^{2}=\frac{v^{2}}{r^{2}}\left(1-\frac{\delta^{2}}{(R-r{)}^{2}}\right)+\frac{2g}{r(1+j)},\;\omega_{30}=-\frac{v\delta }{r(R-r)} & \end{array}$$

since $\dot{\beta }=\omega_{1}$ from equation (3.47). We substitute equation (7.1) into equation (6.13) and write $F(\omega_{10},0,\omega_{30})\equiv H(\delta ,v)$. The level sets of H(δ,v)=0 are depicted in figure 12. Then, because of the symmetry of the governing equations, the golf ball can emerge from a hole of the minimum allowed depth H=0.102m (see appendix A) with initial conditions in a tiny part of region 4 of figure 7, close to $P_{13}$ where $v=c_{1,3}(\delta )$ intersects.

From numerical work in [4, fig. 7], we know that the curve $v=c_{3}(\delta )$ of spin-free rim motion hardly changes when the rim equations with zero initial spin are used (and we have verified that the curve $v=c_{1}(\delta )$ is similarly only slightly modified). Hence, if we select parameters v,δ close to $P_{13}$, we might find a hole lip out if we use the rim equations with zero initial spin. Taking vδ=0.026 and v=0.68, we have δ=0.038 and $\frac{\delta }{R}=0.71$, which is in region 4 of figure 7. Integrating the rim equations with zero initial spin, we find that the golf ball does indeed reach the hole, with $t_{out}=0.11$ and $\omega_{10}=-10.45$, $\omega_{20}=58.25$, $\omega_{30}=-24.37$. Then from equation (6.13), we find F(−10.45,58.25,−24.37)=−0.003<0. So the golf ball will not touch the bottom of the hole, and we should expect a hole lip out.

**Figure 13.**
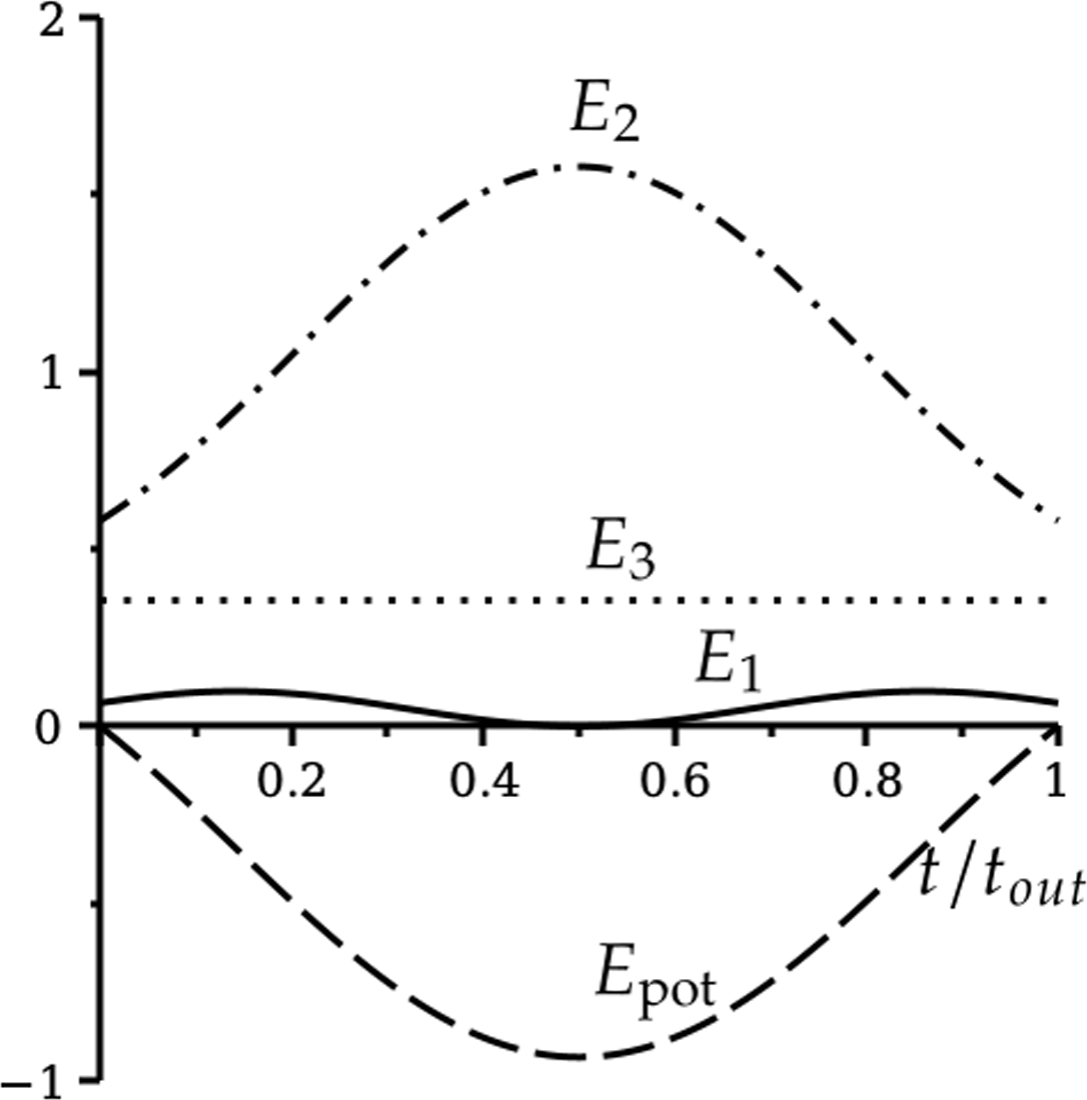
Hole lip out: relative energies $E_{1,2,3,\text{pot}}$ defined in equation (3.55) for the hole motion, plotted as a function of $t/t_{out}$, where $t_{out}=0.51$ is the time spent in the hole, with initial conditions $\omega_{10}=-10.45$, $\omega_{20}=58.25$, $\omega_{30}=-24.37$. Solid line: $E_{1}$. Dash-dotted line: $E_{2}$. Dotted line: $E_{3}$. Dashed line: $E_{\text{pot}}$.

In figure 13, we show the relative energies $E_{1,2,3,\text{pot}}$ defined in equation (3.55) for the hole motion, plotted as a function of $t/t_{out}$, where $t_{out}$ is the time spent in the hole, with initial conditions $\omega_{10}=-10.45$, $\omega_{20}=58.25$, $\omega_{30}=-24.37$. The (relative) energy due to rolling $E_{3}$ is constant, since $\omega_{30}$ is constant. The (relative) energy due to pitching $E_{1}$ varies a little bit, with $\omega_{1}$ changing sign at the minimum of the hole lip out. But the significant observation is that, as the ball falls further into the hole losing potential energy $E_{pot}$, the (relative) energy due to spinning $E_{2}$ is the main beneficiary. Then, as the golf ball returns to the rim with the change in sign in $\omega_{1}$, the energy transfer is reversed.

## 8. Discussion and conclusion

We have presented results on the motion of a golf ball on a putting green as it moves around the rim of the hole and within the hole itself. With the help of a significant number of analytic results, we have shown the existence of two different types of lip out, thus combining in a unified model earlier approaches [4,6–8], which considered them separately. The *rim lip out* happens when the golf ball, moving on the rim of the hole, does not enter the hole (centre of mass stays above the level of the green). Pitching induced by the initial conditions at the rim is not enough to overcome rolling. At the heart of this phenomenon is a family of degenerate saddle equilibrium of the rim governing equations (*the golf balls of death*), where the golf ball rotates around the rim at fixed angle $\beta_{e}$, and fixed angular velocity $\omega_{3}(\beta_{e})$, equation (4.2). A perturbation can take the golf ball either back onto the green or into the hole. In the case of spin-free motion, either the golf ball experiences a rim lip out or it falls directly into the hole, producing a successful putt.

In the presence of spin, the saddle nature of the rim motion persists. But it is spin in the hole motion that allows for a second type of lip out: the *hole lip out*. Here, the golf ball falls into the hole, where it undergoes a pendulum-like motion, as it rolls around the wall of the hole. Its potential energy is converted into spin and then, provided the golf ball does not touch the bottom of the hole, back again as the pitch changes sign. The golf ball returns to the rim and goes back onto the green. This behaviour happens for a very small set of (δ,v) initial conditions.

Hubbard & Smith [4] did not consider the hole motion. Working with the Newton–Euler equations rather than the energy, they established numerically that a saddle was present in the rim equations with spin. But they claimed that the curve $c_{3}(\delta )$ ‘separating the regions of escape and capture does not correspond^30^ to initial conditions $\omega_{1},\omega_{3}$ which evolve to exact equilibrium solutions and thus this boundary is not part of a separatrix’.

Pujol & Pérez [6,7] considered hole motion but did not consider the equations of rim motion. Based on approximations of the motion at the rim, they obtained criteria for hole lip outs that are not directly comparable to ours.

We cannot justify a full sweep of (v,δ,ε) parameter space to establish the boundaries of hole lip outs, because the hole may, or may not, contain a lining, of unknown internal diameter, whose depth within the hole depends on the nature of the soil,^31^ whose properties we cannot know in general. The presence of the lining could interfere with the trajectory of the golf ball in the hole.

We have not considered what we call *ballistic lip outs*^32^. These can occur when the golf ball impacts the opposite rim, rolls along it and then returns to the green. Holmes [8, fig. 12] has a region labelled ER2 (which lies in region 1 of figure 7) in which the golf ball escapes ‘rolling after striking the opposite rim’. To analyse such behaviours requires a different approach, including estimating the time of free-fall, and more importantly, modelling how the roll/slip motion evolves during impact (which may or may not be elastic; see also [10]).

## Data Availability

We have uploaded code that creates the relevant figures in the paper. Supplementary material is available online [24].

## Footnotes

^1^
The lip out is also called a *roll-out* [4], the golfer’s dilemma [5], the golf ball paradox [6] and the golfer’s curse [7]. There are a large number of lip out videos available online, for example, https://www.youtube.com/watch?v=5sbM2Isx17A and https://www.youtube.com/watch?v=4er2buINHF0. Some unfortunate golfers can be seen making three successive lip outs at the same hole.

^2^
The moment of inertia of a sphere of mass *m* and radius *r* can always be written as $jmr^{2}$, whether the sphere is uniform or not. But the coefficient *j* of a non-uniform sphere can be greater than, equal to or less than that of a uniform sphere, depending on how the density of the material varies with radius. Our analytic results are for general *j* (that is, whether the sphere is uniform or non-uniform). We observe no significant dependence on *j*, apart from numerical factors. Our numerical results with j=2/5 can therefore be used as an approximation when a golf ball is non-uniform. Alternatively, our general results can be used if the value of *j* is known for the particular non-uniform golf ball under consideration.

^3^
We do not need the equations for ground motion equations in unified variables. They are given in appendix C for reference.

^4^
In the sequel, we abuse notation, writing either $\omega_{i}(\beta )$ or $\omega_{i}(t)$ as necessary. The context should make clear the dependence.

^5^
The limit μ→∞ in equation (3.27) gives this non-negative condition for $F_{2}$.

^6^
Note that [4] did include a torque that was assumed to be proportional to $F_{2}$.

^7^
Coupling between friction force and torque could also be considered (see, for example, [20–22]), which is not included in the present paper. In that case equation (3.41) would be replaced by a single condition.

^8^
For sufficiently large contact stiffness and low normal forces, $\eta (F_{2})$—and thus $M_{2}$—is negligible, leading to the original model with spin. However, the value of $M_{2}$ can be significant if the typical size of the contact area of the golf ball with the ground is in the same order of magnitude as the size of the ball. Since the stiffness of the grass or soil is not high, it is realistic to expect an (equivalent) contact region size of several millimetres.

^9^
There is a symmetry under reflection in the set $\omega_{2}=0,\;\omega_{3}=0$, and a time-reverse symmetry under reflection in the set $\omega_{1}=0$.

^10^
Later, we will see that we should take the minus sign in equation (4.2). Note that the $\omega_{3e}$ diverges when $\beta_{e}=0$. However, we shall show in the next subsection that this value of $\beta_{e}$ cannot be reached.

^11^
This steady-state motion has similarities to that of motorcycles performing on the *wall of death*, sometimes seen in fairgrounds.

^12^
For $\mu <\mu_{\text{min}}$, slip can occur for intermediate angles around $\beta_{e}=\arccos\sqrt{7/12}=0.70\approx 40.2^{\circ }$.

^13^
The minus sign that occurs in equation (4.10) is the reason we choose the minus sign in equation (4.2).

^14^
Initially, we obtained equation (4.11) via integration of the equations of motion equations (3.44), (3.45) and (3.47). But it is a lengthy and inelegant process.

^15^
There is a similarity between figure 5 and the phase-plane diagram of the inverted pendulum.

^16^
We eschew non-dimensionalization because of the following reasons: (i) previous authors [4,8] have worked in the $\left(\frac{\delta }{R},v\right)$-plane, and so do we for ease of comparison; (ii) a natural choice of scaling for v is $\sqrt{gr}$, but subsequent formulae then involve $\frac{r}{R}=0.40$; and (iii) the radius of the hole R is fixed by the rules of golf (appendix A) and the vast majority of golf balls have the same radius r.

^17^
The further fate of the golf ball is beyond the scope of this paper; one must analyse free-fall, impacts and possible re-attachments with the hole or rim. In the numerical simulations of Holmes [8, fig. 12], seven different outcomes were found in region 1.

^18^
These simulations used the governing equations for the rim motion with spin equations (3.28)–(3.32), assuming zero initial spin, rather than the equations of spin-free motion equations (3.44)–(3.47). The difference is not much; see [4, fig. 7], where a portion of the curve $v=c_{3}(\delta )$ is plotted for the different sets of equations.

**^19Equation (3.49)^ :**gives a contradiction.

^20^
By continuity, they are also the values of $\omega_{1,3}$ at the end of the spin-free rim motion.

^21^
Note that [19, eq. (48)] reduces to equation (5.3) when the radius of the basketball rim is taken to be zero, and that [8, eq. (17)] is identical to equation (5.4), for the golf ball when j=2/5.

^22^
Although equation (5.4) does reduce to equation (4.2) when $\omega_{2e}=0$, the same is not true for the expressions for the eigenvalue equations (5.6) and (4.4), because the dynamics of the motion with spin is in a higher-dimensional space compared with the spin-free motion.

^23^
Note that [19, eq. (54)] reduces to equation (5.6) when the radius of the basketball rim is taken to be zero.

^24^
In [19], there are steady motions where the basketball can separate from the rim because of the larger interval of β.

^25^
In the text, the value $\frac{\delta }{R}=0.89$ is quoted, whereas both captions of [4, figs 4, 5] state that $\frac{\delta }{r}=0.89$. We get much better agreement with the value in the text.

^26^
Note that we reset time again in this section, so that the golf ball enters the hole z=0 at t=0.

^27^
Ω is directly related to the eigenvalue equation (5.12) of the steady-state hole solution $x_{eh}$ in equations (5.10) and (5.11).

^28^
We get the same result if we divide equation (3.35) by (3.38) and integrate both sides with respect to β.

^29^
The golf ball is then subject to the rim equations again.

^30^
since ‘. . . it still would take infinite time to arrive at the equilibrium . . . ’.

^31^
See appendix A.

^32^
An example of a ballistic lip out appears to be given at https://youtube.com/shorts/ovG63teINPI?feature=shared.

## Authors' Notes

S.J.H. is not related, unfortunately, to one of the best golfers of all time, Ben Hogan (1912–1997).

## Appendix A. Extract from the rules of golf

From the rules of golf (https://www.randa.org/rules/rules-hub), ‘Rule 4.2: The weight of the ball must not be greater than 1.620 ounces avoirdupois (45.93 g). There is no minimum weight thus a ball can be as light as the manufacturer desires’.

‘Rule 4.3 The diameter of the ball must not be less than 1.680 inches (42.67 mm). It is important to note that there is no maximum size, the ball can be as large as desired provided it conforms to all other standards’.

Elsewhere, ‘The hole must be 4¼ inches (108 mm) in diameter and at least 4 inches (101.6 mm) deep. If a lining is used, its outer diameter must not exceed 4¼ inches (108 mm). The lining must be sunk at least 1 inch (25.4 mm) below the putting green surface, unless the nature of the soil requires that it be closer to the surface’.

## Appendix B. Comparison between golf ball and basketball equations

Since [4] assumes that the golf ball rolls without slipping, we set the components of the velocity of the contact point $u_{1}=u_{3}=0$ in [19]. In addition, [4] assumes a Coulomb friction torque $\tau_{r}$, which is absent from [19]. So we assume $\tau_{r}=0$ in [4]. Then the equations in the two papers can be derived from one another (table 1).

**Table 1.** Equivalence of equations between [4] and [19] when $u_{1}=u_{3}=0$ and $\tau_{r}=0$.

basketball [19]	golf ball [4]	meaning
(16)	(1)	angular velocity of ball
(13)	(3)	velocity of ball
(19)	(5)	kinematic constraint
(20)	(6)	kinematic constraint
(35)	(8)	derivative of angular velocity
(34)	(9)	derivative of angular velocity
(33)	(10)	derivative of angular velocity

## Appendix C. Ground motion in unified variables

Although of no practical use for our purposes, it is possible to express the *ground motion* in terms of unified variables, corresponding to β∈[π/2,π). Then ρ=−R+rcot⁡β,z=r and $\beta^{*}=\pi /2$. The time derivatives of α and β are given by $\dot{\alpha }=\frac{-\omega_{3}r}{R-r\;\cot\;\beta }$, $\dot{\beta }=\omega_{1}\sin^{2}\;\beta$.

The equations of the ground motion are

$$\begin{array}{c} {\dot{\omega }}_{1}=\frac{\omega_{3}^{2}r}{R-r\;\cot\;\beta },\;{\dot{\omega }}_{2}=0,\;{\dot{\omega }}_{3}=\frac{-r\omega_{1}\omega_{3}}{R-r\;\cot\;\beta },\;\dot{\alpha }=\frac{-r\omega_{3}}{R-r\;\cot\;\beta },\;\dot{\beta }=\omega_{1}\sin^{2}\;\beta . \end{array}$$

The contact forces of the ground motion are

$$\begin{array}{c} F_{1}=0,\;F_{2}=mg,\;F_{3}=0. \end{array}$$

The solution $x_{eg}=(\beta_{e},0,\omega_{2e},0{)}^{T}$ for constant $\beta_{e},\;\omega_{2e}$ is the steady state of the ground motion. It corresponds to the golf ball rotating about the vertical, with arbitrary fixed angular velocity $\omega_{2e}$, at an arbitrary distance $d=-r\;\cot\;\beta_{e}$ from the rim edge; note that d≥0 since $\beta_{e}\in \left[\frac{\pi }{2},\pi \right)$. We can also show that

$$\begin{array}{c} \omega_{3}(\beta )=\frac{A_{g}\tan\;\beta }{R\;\tan\;\beta -r},\;\omega_{1}^{2}(\beta )=2rA_{g}^{2}\left(B_{g}-\frac{1}{2r(R-r\;\cot\;\beta {)}^{2}}\right), \end{array}$$

where $A_{g},B_{g}$ are constants of integration.

## Appendix D. Derivation of equations (3.25) and (3.26)

From §3.2, the Newton–Euler equations (retaining the same equation numbers) of the golf ball are given by

(3.23)maG=FC+FG,(3.24)jmr2ε=rGC×FC,

where the gravitational force $F_{G}$ is given by

$$\begin{array}{lcr} & F_{G}=-mg\;\sin\;\beta^{*}g_{2}-mg\;\cos\;\beta^{*}g_{3}, & \text{(3.21)} \end{array}$$

the force $F_{C}$ acting at the contact point is given by

$$\begin{array}{lcr} & F_{C}=F_{1}g_{1}+F_{2}g_{2}+F_{3}g_{3}, & \text{(3.22)} \end{array}$$

the acceleration $a_{G}$ of G is given by

$$\begin{array}{lcr} & a_{G}=\left(-{\dot{\omega }}_{3}r-\dot{\alpha }\dot{\rho }\right)g_{1}+\left(-\omega_{1}{\dot{\beta }}^{*}r-{\dot{\alpha }}^{2}\rho \;\cos\;\beta^{*}\right)g_{2}+\left({\dot{\omega }}_{1}r+{\dot{\alpha }}^{2}\rho \;\sin\;\beta^{*}\right)g_{3}, & \text{(3.17)} \end{array}$$

and the angular acceleration $\varepsilon =\dot{\omega }$ is given by

ε=ω˙=(ω˙1+ω3α˙sin⁡β∗−ω2α˙cos⁡β∗)g1+(ω˙2−ω3β˙∗+ω1α˙cos⁡β∗)g2(3.18)+(ω˙3+ω2β˙∗−ω1α˙sin⁡β∗)g3.

Using equations (3.18), (3.21) and (3.22), the components of equation (3.24) become

(D1)
$$\begin{array}{rl} jmr^{2}({\dot{\omega }}_{1}+\omega_{3}\dot{\alpha }\sin\beta^{*}-\omega_{2}\dot{\alpha }\cos\beta^{*}) & =-rF_{3},\\ jmr^{2}({\dot{\omega }}_{2}-\omega_{3}{\dot{\beta }}^{*}+\omega_{1}\dot{\alpha }\cos\beta^{*}) & =0,\\ jmr^{2}({\dot{\omega }}_{3}+\omega_{2}{\dot{\beta }}^{*}-\omega_{1}\dot{\alpha }\sin\beta^{*}) & =rF_{1}. \end{array}$$

The second of these equations is equation (3.25)${,}_{2}$. Using equations (3.17), (3.21) and (3.22), the components of equation (3.23) become

(D2)
$$\begin{array}{rl} m(-{\dot{\omega }}_{3}r-\dot{\alpha }\dot{\rho }) & =F_{1},\\ m(-\omega_{1}{\dot{\beta }}^{*}r-{\dot{\alpha }}^{2}\rho \cos\beta^{*}) & =-mg\sin\beta^{*}+F_{2},\\ m({\dot{\omega }}_{1}r+{\dot{\alpha }}^{2}\rho \sin\beta^{*}) & =-mg\cos\beta^{*}+F_{3}. \end{array}$$

Eliminating $F_{3}$ from both sets of equations and using equation (3.12)${,}_{1}$ gives equation (3.25)${,}_{1}$; eliminating $F_{1}$ and using equation (3.12)${,}_{2}$ gives equation (3.25)${,}_{3}$. Hence, we have the derivation of equation (3.25).

In the equation for $F_{1}$ in (D2), we substitute for ${\dot{\omega }}_{3}$ from equation (3.25)${,}_{3}$, and for $\dot{\rho }$ from equation (3.12)${,}_{2}$ to get equation (3.26)${,}_{1}$. The second equation in (D2) is equation (3.26)${,}_{2}$. In the equation for $F_{3}$ in (D2), we substitute for ${\dot{\omega }}_{1}$ from equation (3.25)${,}_{1}$, and for $\dot{\alpha }$ from equation (3.12)${,}_{1}$ to get equation (3.26)${,}_{3}$. Hence, we have the derivation of equation (3.26).

## Appendix E. Vertical tangent and asymptote in figure 8

The curve $h(\beta_{e},\omega_{3e})=0$ equation (5.6) has a vertical tangent in the $\left(\beta_{e},\omega_{3e}\right)$-plane when $\frac{\partial h}{\partial \omega_{3e}}=0$, when

$$\begin{array}{c} \omega_{3e}^{2}=g\frac{(R-r\;\cos\;\beta_{e}{)}^{\frac{3}{2}}}{r^{2}}\sqrt{\frac{\cos\;\beta_{e}}{\left(1+j)[R\;\cos\;\beta_{e}-(1+j)r\right]}}, \end{array}$$

and where $\beta_{e}$ is given by

(E 1)
$$\begin{array}{lcr} & r^{2}(1+j)\;\cos^{3}\;\beta_{e}-rR\;\cos^{2}\;\beta_{e}+R^{2}\cos\;\beta_{e}-rR(1+j)=0. & \end{array}$$

Note that [19, eq. (55)] reduces to equation (E1) when the radius of the basketball rim is zero. We solve equation (E1) for $\cos\;\beta_{e}$, and find the corresponding value(s) of $\omega_{3e}^{2}$. The discriminant Δ of equation (E1) is given by

$$\begin{array}{c} \Delta =R^{2}r^{2}[(-4j-3)R^{4}+2(9j+7)(1+j)R^{2}r^{2}-27(1+j{)}^{4}r^{4}]. \end{array}$$

It is straightforward to show that Δ<0 for the parameters of the golf ball, and so the cubic equation (equation (E1)) has only one real root, $\cos\;\beta_{e}=0.66$, corresponding to $\beta_{e}=0.85=48.43^{\circ }$. Hence, $\omega_{3e}(\beta_{e})=-39.09$ and from equation (5.3) $\omega_{2e}(\beta_{e})=-99.38$. The vertical asymptote to the curve $h(\beta_{e},\omega_{3e})=0$ occurs at $\beta_{e}=0.98=56.40^{\circ }$.
